# Representative Points of the Inverse Gaussian Distribution and Their Applications

**DOI:** 10.3390/e27121190

**Published:** 2025-11-24

**Authors:** Wen-Wen Hu, Kai-Tai Fang, Xiao-Ling Peng

**Affiliations:** 1 Faculty of Science and Technology, Beijing Normal-Hong Kong Baptist University, Zhuhai 519087, China; wenwenhu@bnbu.edu.cn (W.-W.H.); ktfang@bnbu.edu.cn (K.-T.F.); 2 The Key Lab of Random Complex Structures and Data Analysis, The Chinese Academy of Sciences, Beijing 100045, China; 3 Guangdong Provincial Key Laboratory of Interdisciplinary, Research and Application for Data Science (IRADS), Beijing Normal-Hong Kong Baptist University, 2000 Jintong Road, Zhuhai 519088, China

**Keywords:** inverse gaussian distribution, parameter estimation, representative points

## Abstract

The inverse Gaussian (IG) distribution, as an important class of skewed continuous distributions, is widely applied in fields such as lifetime testing, financial modeling, and volatility analysis. This paper makes two primary contributions to the statistical inference of the IG distribution. First, a systematic investigation is presented, for the first time, into three types of representative points (RPs)—Monte Carlo (MC-RPs), quasi-Monte Carlo (QMC-RPs), and mean square error RPs (MSE-RPs)—as a tool for the efficient discrete approximation of the IG distribution, thereby addressing the common scenario where practical data is discrete or requires discretization. The performance of these RPs is thoroughly examined in applications such as low-order moment estimation, density function approximation, and resampling. Simulation results demonstrate that the MSE-RPs consistently outperform the other two types in terms of approximation accuracy and robustness. Second, the Harrell–Davis (HD) and three Sfakianakis–Verginis (SV1, SV2, SV3) quantile estimators are introduced to enhance the representativeness of samples from the IG distribution, thereby significantly improving the accuracy of parameter estimation. Moreover, case studies based on real-world data confirm the effectiveness and practical utility of this quantile estimator methodology.

## 1. Introduction

Statistical distributions hold a pivotal position in information theory, as they outline the probabilistic features of data or signals, thereby directly influencing the precision and effectiveness of information representation, transmission, compression, and reconstruction. Entropy, being the foremost metric in the realm of information theory, relies on the statistical distribution of the random variable. Numerous applications in information theory necessitate the presumption of the data’s statistical distribution. While the normal distribution is commonly adopted in most statistical analyses owing to its mathematical ease and broad applicability, real-world data often display skewness, prompting the need for more adaptable models. Brownian motion is a widely used model for stochastic processes. In 1915, Schrödinger [1] described the probability distribution of the first passage time in Brownian motion. Thirty years later, in 1945, Tweed [2] gave the inverse relationship between the cumulant generating function of the first passage time distribution and that of the normal distribution, and named it the inverse Gaussian (IG) distribution. Then, in 1947, Wald [3] derived the distribution as a limiting form for the sample size distribution in a sequential probability ratio test, leading to it being known in Russian literature as the Wald distribution. IG distribution is discussed in various books on stochastic processes and probability theory, such as Cox and Miller [4] and Bartlett [5]. This distribution is also known as the Gaussian first passage time distribution [6], and sometimes as the first passage time distribution of Brownian motion with positive drift [7]. IG distribution is suitable for modeling asymmetric data due to its skewness and relationship to Brownian motion. Folks and Chhikara [8] and Chhikara and Folks [9] have conducted a comprehensive examination of the mathematical and statistical properties of this distribution.

The interpretation of the inverse Gaussian random variable as a first passage time indicates its potential usefulness in examining lifetime or the frequency of event occurrences across various fields. Chhikara and Folks [10] suggested that IG distribution is a useful model for capturing the early occurrence of events like failures or repairs in the lifetime of industrial products. Iyengar and Patwardhan [11] indicated the possibility of using IG distribution as a useful way to model failure times of equipment. The IG distribution has found applications in various fields. For example, Kourogiorgas et al. [12] applied IG distribution to a new rain attenuation time series synthesizer for Earth–space links, which enables accurate evaluation of how satellite communication networks are operating. In insurance and risk analysis, Punzo [13] used IG distribution to model bodily injury claims and to analyze economic data concerning Italian households’ incomes. In traffic engineering, Krbálek [14] used IG distribution models for vehicular flow.

In statistics, for an unknown continuous statistical distribution, using an empirical distribution of random samples is a conventional approach to approximate the target distribution. Nevertheless, this method frequently results in low accuracy. Thus, to retain as much information of the target distribution as possible, the support points for the discrete approximation, also called representative points (RPs), are investigated. For a comprehensive review of RPs, one may refer to Fang and Pan [15]. Representative points hold significant potential for applications in statistical simulation and inference. One promising application is a new approach to simulation and resampling that integrates number-theoretic methods. Li et al. [16] propose moment-constrained mean square error representative samples (MCM-RS), which are generated from a continuous distribution via a method that minimizes the mean square error (MSE) between the sample and the original distribution while ensuring the first two sample moments match preset values. Peng et al. [17] utilized representative points from a Beta distribution to weight patient origin data, thereby constructing the Patient Regional Index (PRI). This study serves as a practical application of representative points from different distributions. In the existing literature, various types of representative points corresponding to different statistical distributions have been investigated. Especially for complex distributions, the study of their representative points is indispensable. Many authors investigated the problem of discretizing a MixN by a fixed number of points under the minimum mean squared error, MSE-RPs of Pareto distributions, their estimation, and RPs of generalized alpha skew-t distribution with applications [18]. To the best of our knowledge, the representative points of the inverse Gaussian distribution have not yet been investigated, despite their potential utility. Therefore, we investigate three types of representative points for the inverse Gaussian distribution and explore their applications in estimation of moments and density function, as well as resampling.

This paper aims to investigate the applications of RPs as well as a dedicated parameter estimation approach based on nonparametric quantile estimators for the IG distribution. The main contributions of this work are summarized as follows:

1. We establish, to the best of our knowledge, the first systematic comparative framework for three distinct types of representative points—Monte Carlo (MC-RPs), quasi-Monte Carlo (QMC-RPs), and mean square error RPs (MSE-RPs)—specifically on the Inverse Gaussian distribution. This framework provides a benchmark for evaluating discrete approximation quality in terms of moment estimation, density approximation, and resampling efficiency.
2. We introduce a novel parameter estimation methodology for the IG distribution by employing the Harrell–Davis (HD) [19] and Sfakianakis–Verginis (SV1, SV2, SV3) [20] quantile estimators. This constitutes the first application and comprehensive demonstration of these estimators for enhancing sample representativeness and significantly improving the accuracy of IG parameter estimation.
3. Through extensive simulations and real-world case studies, we provide a comprehensive performance analysis. Our results not only conclusively demonstrate the superiority of MSE-RPs in approximation tasks but also validate the practical utility and effectiveness of the proposed quantile-based estimation framework.

The rest of this paper is organized as follows. Section 2 introduces the fundamental properties of the inverse Gaussian distribution. Section 3 details the generation of representative points and their applications in statistical simulation. Section 4 analyzes resampling methods based on representative points for the IG distribution. Section 5 discusses parameter estimation for the IG distribution using samples enhanced by the introduced nonparametric quantile estimators. A real-data case study is presented in Section 6. Finally, Section 7 provides the conclusions and future research directions.

## 2. Basic Properties of the IG Distribution

**Definition** **1.** *Consider a random variable X to follow IG distribution IG(μ,λ), denoted as X∼IG(μ,λ). The probability density function (pdf) of X is defined as*

(1) $$\begin{array}{c} f\left(x;\mu ,\lambda \right)=\sqrt{\frac{\lambda }{2\pi x^{3}}}\exp\left\{\frac{-\lambda {\left(x-\mu \right)}^{2}}{2\mu^{2}x}\right\}, \end{array}$$

*where* x>0, μ>0, and λ>0. *Denote its distribution function by*$F_{\mathrm{IG}}\left(x;\mu ,\lambda \right)$.

The mean of the IG distribution is μ, and the variance is $\mu^{3}/\lambda$ [21]. The maximum likelihood estimates (MLEs) of μ and λ are

(2) $$\begin{array}{c} {\hat{\mu }}_{ML}=\bar{X}=\sum_{i=1}^{n}X_{i}/n,\;{\hat{\lambda }}_{ML}=n/\sum_{i=1}^{n}\left(1/X_{i}-1/\bar{X}\right), \end{array}$$

where $X_{1},\ldots ,X_{n}$ is a random sample from IG(μ,λ). Furthermore, it is also known that $\bar{X}∼IG\left(\mu ,n\lambda \right)$, $\lambda \sum_{i=1}^{n}\left(1/X_{i}-1/\bar{X}\right)∼\chi_{n-1}^{2}$, and $\bar{X}$ and $\lambda \sum_{i=1}^{n}\left(1/X_{i}-1/\bar{X}\right)$ are independent [22]. Folks and Chhikara [8] proved that the uniformly minimum variance unbiased estimators for μ and λ are

(3) $$\begin{array}{c} {\hat{\mu }}_{UMVUE}=\bar{X},\;{\hat{\lambda }}_{UMVUE}=\left(n-3\right)/\sum_{i=1}^{n}\left(1/X_{i}-1/\bar{X}\right). \end{array}$$

**Figure 1:**
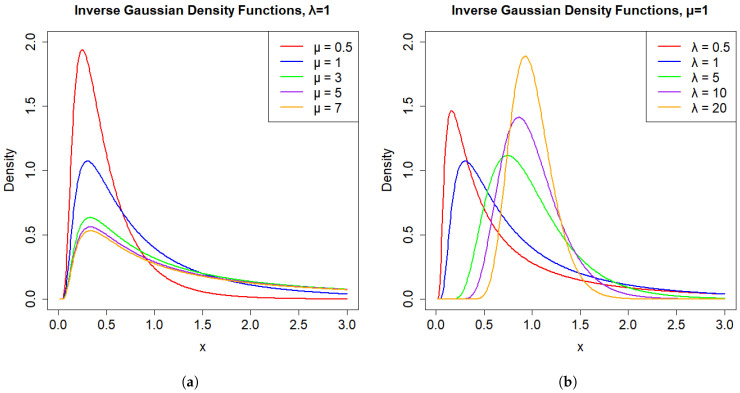
(**a**) Density functions of IG(μ,λ) for fixed λ and increasing μ. (**b**) Density functions of IG(μ,λ) for fixed μ and increasing λ.

The shapes of the IG distributions with varying parameter sets are depicted in Figure 1. For Figure 1a, when μ is fixed and for the lower values of λ, the distribution demonstrates a higher peak and a steep decline in probability, which suggests a sharper distribution. As λ increases, the peak becomes less pronounced, indicating a distribution with a heavier tail. From Figure 1b, it can be seen that as μ increases, the peak value of the density curve gradually decreases, and the peak position moves along the positive direction of the *x*-axis. At the same time, the entire curve extends to the right, reflecting the influence of the mean μ of the inverse Gaussian distribution on the distribution position and shape. The larger μ is, the more to the right the center of the distribution is, and the flatter the peak is.

## 3. Representative Points of IG Distribution

Three types of representative points will be discussed in this section, namely MC-RPs, QMC-RPs, and MSE-RPs, from the parametric k-means algorithm, the NTLBG algorithm, and obtained by solving a system of nonlinear equations (the Fang–He algorithm [23]) of the IG(μ,λ). Furthermore, we also discuss the applications of representative points, which are applied to moment estimation and density estimation.

### 3.1. Three Types of Representative Points

#### 3.1.1. MC-RPs

Let X∼F(x;θ) be a random vector, where θ denotes the parameters. In conventional statistics, inferences about the population are drawn using an independent and identically distributed random sample $x_{1},\ldots ,x_{k}$ from *F*. The empirical distribution is defined as

(4) $$\begin{array}{c} F_{k}\left(x\right)=\frac{1}{k}\sum_{i=1}^{k}I_{\left\{x_{i}\leq x\right\}}, \end{array}$$

where $I_{A}$ is the indicator function of set *A*. This is a discrete distribution assigning probability 1/k to each sample point, serving as a consistent approximation to F(x).

In statistical simulation, let $Y_{\mathrm{MC}}$ denote the random samples generated computationally via Monte Carlo methods; we denote this as $Y_{\mathrm{MC}}∼F_{k}\left(y\right)$. Efron [24] extended this idea into the bootstrap method, where samples are drawn from the empirical distribution $F_{k}$ rather than *F*.

Although widely used, the MC method has limited efficiency due to the slow convergence rate $O_{p}\left(1/\sqrt{k}\right)$ of $(F_{k}\left(x\right)$ to F(x). This slow convergence leads to suboptimal performance in numerical integration, motivating alternative approaches.

#### 3.1.2. QMC-RPs

Consider computing a high-dimensional integral in the canonical form:

(5) $$\begin{array}{c} I\left(g\right)=\int_{0}^{1}\cdots \int_{0}^{1}g\left(x_{1},\ldots ,x_{d}\right)dx_{1}\ldots dx_{d}=\int_{C^{d}}g\left(x\right)dx, \end{array}$$

where *g* is a continuous function defined on $C^{d}={\left[0,1\right]}^{d}$. Monte Carlo methods approximate I(g) using random samples from $U(C^{d})$, achieving a convergence rate of $O_{p}\left(1/\sqrt{k}\right)$. Quasi-Monte Carlo (QMC) methods improve this by constructing point sets $Y=\{y_{1},\ldots ,y_{k}\}$ that are evenly dispersed over $C^{d}$, achieving a convergence rate of $O(k^{-1}{\left(\log k\right)}^{d})$. For the theoretical foundations and methodologies of QMC, one may consult works by Hua and Wang [25] as well as Niederreiter [26]. In earlier research, the star discrepancy was frequently used by many scholars as a metric to assess the uniformity of Y within $C^{d}$. The star discrepancy is defined as

(6) $$\begin{array}{c} D\left(G,G^{\left(k\right)}\right)=\underset{x\in R^{d}}{\sup}\left|G\left(x\right)-G^{\left(k\right)}\left(x\right)\right|, \end{array}$$

where G(x) represents the cumulative distribution function (cdf) of $U(C^{d})$ and $G^{\left(k\right)}\left(x\right)$ is the empirical distribution corresponding to Y. An optimal set Y minimizes $D(G,G^{\left(k\right)})$. In such a case, the points in Y are termed QMC-RPs, which serve as support points of $G^{\left(k\right)}\left(y\right)$ with each point having an equal probability of 1/k.

The optimality of the point set $\left\{q_{i}\right\}=\left\{\left(2i-1\right)/\left(2k\right)\right\}$ has a profound theoretical foundation. As demonstrated in Examples 1.1 and 1.3 of the seminal work by Fang and Wang [27], this set achieves the lowest star-discrepancy, establishing its optimality under a prominent criterion in quasi-Monte Carlo methods for any continuous distribution. Meanwhile, from the perspective of statistical distance minimization, Barbiero and Hitaj [28] presented the following fundamental result, proving that the same point set is also optimal under the Cramér–von Mises criterion.

**Theorem** **1.** *Let* F(x) *be a strictly increasing and continuous cumulative distribution function, and k be a positive integer. Consider the set* $F_{k}$ *of all discrete distributions with k support points, with cumulative distribution function* $\hat{F}\left(x\right)$.

*Then, for any r>0, the unique optimal discrete distribution $\hat{F}\in F_{k}$ that minimizes the Cramér–von Mises distance family*

$$d_{r}\left(F,\hat{F}\right)=\int_{0}^{1}{\left|t-\hat{F}\left(F^{-1}\left(t\right)\right)\right|}^{r}\;dt$$

*is given by the following:*

*Support Points (Quantization Points): $x_{i}=F^{-1}\left(q_{i}\right)$, where $q_{i}=\frac{2i-1}{2k}$, for i=1,…,k.*

*Probabilities (Weights):$p_{i}=\frac{1}{k}$, for i=1,…,k (i.e., a discrete uniform distribution).*

In this paper, we construct the QMC-RPs for the distribution $F_{\mathrm{IG}}\left(x;\mu ,\lambda \right)$ by applying the above theoretical result. Specifically, the support points are generated via the inverse transformation method using the optimal point set

(7) $$\begin{array}{c} b_{j}=F^{-1}\left(\frac{2j-1}{2k}\right),j=1,\ldots ,k, \end{array}$$

with corresponding probability $P\left(Y=b_{j}\right)=\frac{1}{k}$ [16, 32]. Here, $F_{\mathrm{IG}}^{-1}\left(y\right)$ is the inverse function of $F_{\mathrm{IG}}\left(x\right)$, and the set of points $\left\{\frac{2j-1}{2k},j=1,\ldots ,k\right\}$ is uniformly scattered on the interval (0, 1) and, as established, optimal in both the star-discrepancy and Cramér–von Mises senses.

#### 3.1.3. MSE-RPs

MSE-RPs, also known as principal points [29], are representative points designed to minimize the mean square error between the distribution and its discrete approximation. MSE-RPs were independently proposed by Cox [30], Fang and He [23], and many others. For a random variable X∼IG(μ,λ) with density f(x), MSE-RPs are constructed as follows:

Take $-\infty <b_{1}<b_{2}<\ldots <b_{k}<\infty$ and define a stepwise function

(8) $$\begin{array}{c} Q_{b}\left(x\right)=b_{i},\;\;\mathrm{when}\;a_{i}<x⩽a_{i+1},\;i=1,\ldots ,k, \end{array}$$

where $a_{1}=-\infty ,a_{i}=\left(b_{i}+b_{i-1}\right)/2,i=2,\ldots ,k,a_{k+1}=\infty$. Define the mean square error (MSE) to measure bias between F(x) and $Q_{b}\left(x\right)$ as follows:

(9) $$\begin{array}{ccc} \mathrm{MSE}(b)\; & = & \;\mathrm{MSE}\left(b_{1},\ldots ,b_{k}\right)=\frac{1}{\sigma^{2}}E{\left(X-Q_{b}\left(X\right)\right)}^{2}\\ & = & \;\frac{1}{\sigma^{2}}\int_{-\infty }^{+\infty }\min_{i}{\left(x-b_{i}\right)}^{2}f\left(x\right)dx=\frac{1}{\sigma^{2}}\sum_{i=1}^{k}\int_{a_{i}}^{a_{i+1}}{\left(x-b_{i}\right)}^{2}f\left(x\right)dx. \end{array}$$

To find $b^{*}=\left(b_{1}^{*},\ldots ,b_{k}^{*}\right)$, such that MSE(b) arrives at its minimum, the solution of $B^{*}=\left\{b_{1}^{*},\ldots ,b_{k}^{*}\right\}$ is just MSE-RPs of $F_{\mathrm{IG}}\left(x;\mu ,\lambda \right)$. The probability of each representative point is given by

(10) $$\begin{array}{c} f\left(Q_{b}\left(X\right)=b_{i}\right)=p_{i},\;i=1,\ldots ,k, \end{array}$$

where

(11) $$\begin{array}{ccc} p_{1}\; & = & \;\int_{-\infty }^{\frac{b_{1}+b_{2}}{2}}f\left(x\right)dx=\int_{-\infty }^{a_{1}}f\left(x\right)dx,\\ p_{i}\; & = & \;\int_{\frac{b_{i}+b_{i-1}}{2}}^{\frac{b_{i}+b_{i+1}}{2}}f\left(x\right)dx=\int_{a_{i}}^{a_{i+1}}f\left(x\right)dx,\;i=2,\ldots ,k-1,\\ p_{k}\; & = & \;\int_{\frac{b_{k-1}+b_{k}}{2}}^{\infty }f\left(x\right)dx=\int_{a_{k}}^{\infty }f\left(x\right)dx. \end{array}$$

In this paper, we use the following three main different approaches to generate MSE-RPs:

(a) NTLBG algorithm: Combines QMC methods with the k-means-based LBG algorithm for univariate distributions [31].

(b) Parametric k-means algorithm: An iterative method (Lloyd’s algorithm) for finding RPs of continuous univariate distributions [32,33].

(c) Fang–He algorithm: Solves a system of nonlinear equations to find highly accurate MSE-RPs, though computationally intensive for large k [23].

For convenience, we use PKM-RPs, NTLBG-RPs, and FH-RPs to denote RPs from the parametric k-means algorithm, the NTLBG algorithm, and the Fang–He algorithm [23] of the IG(μ,λ), respectively.

### 3.2. Lower Moments Estimation Based on RPs of IG Distribution

In this subsection, we consider estimation of mean, variance, skewness, and kurtosis of X∼IG(μ,λ). QMC-RPs, MSE-RPs which include PKM-RPs, FH-RPs, and NTLBG-RPs mentioned in the previous subsection are used for simulation. To save space in the main text, the specific numerical results of these representative points under different sizes (k=5,10,15,20,25,28,30) are provided in the Appendix A. Readers may refer to the Appendix A for detailed data.

We have obtained mean and variance of X∼IG(μ,λ) in Section 2, E(X)=μ, Var$\left(X\right)=\frac{\mu^{3}}{\lambda }$. Denote the following statistics of *X* by

(12) $$\begin{array}{ccc} \mathrm{Sk}(X) & = & \frac{E{\left(X-\mu \right)}^{3}}{\mathrm{Var}{\left(X\right)}^{\frac{3}{2}}},\;\mathrm{Ku}\left(X\right)=\frac{E{\left(X-\mu \right)}^{4}}{\mathrm{Var}{\left(X\right)}^{2}}-3. \end{array}$$

It can be concluded that

(13) $$\begin{array}{ccc} \mathrm{Sk}(X) & = & 3\sqrt{\frac{\mu }{\lambda }},\;\mathrm{Ku}\left(X\right)=15\frac{\mu }{\lambda }. \end{array}$$

Consider a group of representative points $b=\{b_{1},\ldots ,b_{k}\}$ from IG(μ,λ), where $b_{i}$ with probability $p_{i}$. Then the above statistics are

(14) $$\begin{array}{c} E\left(b\right)=\sum_{i=1}^{k}b_{i}p_{i},\;\mathrm{Var}\left(b\right)=\sum_{i=1}^{k}{\left(b_{i}-\mu_{b}\right)}^{2}p_{i},\\ \mathrm{Sk}\left(b\right)=\frac{1}{\mathrm{Var}{\left(b\right)}^{\frac{3}{2}}}\sum_{i=1}^{k}{\left(b_{i}-\mu_{b}\right)}^{3}p_{i},\;\mathrm{Ku}\left(b\right)=\frac{1}{\mathrm{Var}{\left(b\right)}^{2}}\sum_{i=1}^{k}{\left(b_{i}-\mu_{b}\right)}^{4}p_{i}-3. \end{array}$$

In the following comparisons, we employ MC-RPs, QMC-RPs, FH-RPs, PKM-RPs, and NTLBG-RPs from IG(1, 1) and consider the sample size n=5,10,15,20,25,28,30. Therefore, the corresponding mean, variance, skewness, and kurtosis are 1, 1, 3, and 15. For each statistic (mean, variance, skewness, and kurtosis) there are five estimators and five corresponding biases; Table 1 shows numerical results. It is evident that MC-RPs consist of random samples with size *n*. For the sake of fair comparisons, we generate 10 samples each with size *n*, and then take the average of the estimated statistics as the result of MC-RPs (10).

We have marked the RP methods which have smallest absolute bias in boldface in Table 1. From the results in Table 1 we may raise the following observations: (1) the estimators of FH-RPs and PKM-RPs are more accurate than those of MC-RPs (10), QMC-RPs, and NTLBG-RPs; (2) FH-RPs and PKM-RPs have same performance in estimation of these four statistics. In fact, the bias values in Table 1 are rounded to four decimal places, which makes FH-RPs and PKM-RPs appear to perform identically. However, the performance of FH-RPs and PKM-RPs is not exactly the same. If the bias values are rounded to eight decimal places, it can be observed that FH-RPs performs better than PKM-RPs.

### 3.3. Density Estimation via RPs of IG Distribution

In this section, we estimate density function of inverse gaussian distribution IG(1, 1), choose n=30 as size of the input data, and give comparisons among the four RP methods (QMC-RPs, FH-RPs, PKM-RPs, and NTLBG-RPs). It is noteworthy that for the density estimation based on MC-RPs, owing to the inherent randomness of the Monte Carlo method, the density curve obtained from each fitting process varies significantly. Therefore, the results of MC-RPs-based density estimation will not be presented here.

Rosenblatt [34] and Parzen [35], provides a way to estimate the density function based on a set of samples, $x_{1},\ldots ,x_{n}$. The estimate function

(15) $$\begin{array}{c} {\hat{p}}_{h}\left(x\right)=\frac{1}{n}\sum_{i=1}^{n}k_{h}\left(x-x_{i}\right)=\frac{1}{nh}\sum_{i=1}^{n}ker(\frac{x-x_{i}}{h}) \end{array}$$

is called kernel density estimation, where ker() is the kernel function, *h* is the bandwidth, and $k_{h}\left(y\right)=\frac{1}{h}ker(y/h)$. The most popular kernel is the standard density function; therefore, we choose it as the kernel. In this section, we use a set of representative points with related probabilities to replace *n* i.i.d. samples. In this case, (6) becomes

(16) $$\begin{array}{c} {\hat{p}}_{h}\left(x\right)=\sum_{i=1}^{n}k_{h}\left(x-x_{i}\right)p_{i}=\frac{1}{h}\sum_{i=1}^{n}ker\left(\frac{x-x_{i}}{h}\right)p_{i}, \end{array}$$

and the choice of the bandwidth (h) is very important.

In our experiment, we divide the range of *x* into 2 parts, and choose *h* to have minimum $L_{2}-distance$ between ${\hat{p}}_{h}\left(x\right)$ and p(x) in each part. Now, (7) becomes

(17) $$\begin{array}{c} {\hat{p}}_{h}\left(x\right)=\sum_{i=1}^{n}k_{h}\left(x-x_{i}\right)p_{i}=\sum_{j=1}^{2}\frac{1}{h_{j}}\sum_{i=1}^{n}ker\left(\frac{x-x_{i}}{h_{j}}\right)p_{i}. \end{array}$$

**Figure 2:**
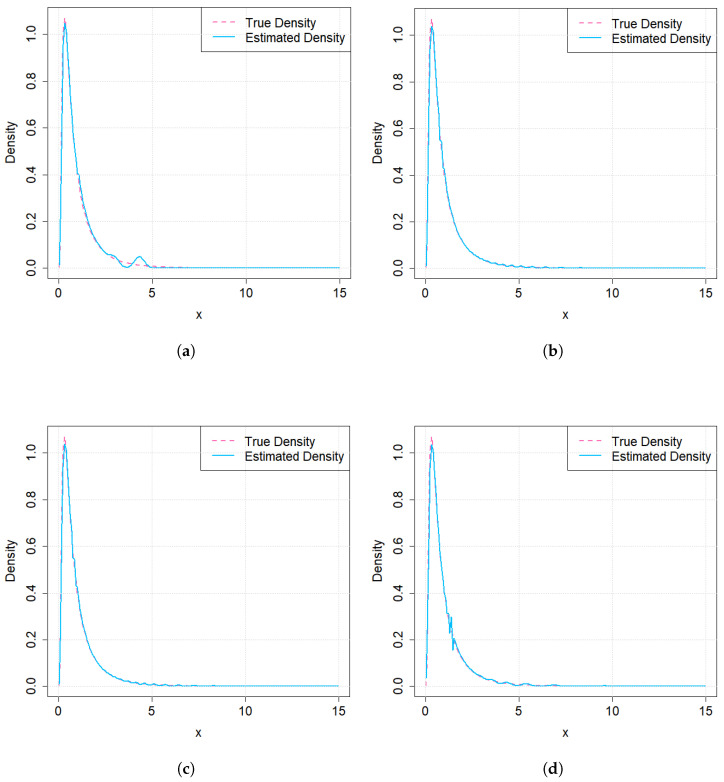
Kernel density estimations from 4 kinds of RPs, when sample size n=30. (**a**) QMC-RPs (**b**) FH-RPs. (**c**) PKM-RPs. (**d**) NTLBG-RPs.

The four density estimators are shown in Figure 2. From Figure 2, it can be observed that FH-RPs and PKM-RPs have similar performance, and their performance is better than that of QMC-RPs and NTLBG-RPs.

The recommended *h* and $L_{2}$-distance between each density estimator and IG(1, 1) in each zone are given in Table 2.

## 4. Resampling Based on RPs of IG Distribution

In this section, we use 4 kinds of RPs (QMC-RPs, FH-RPs, PKM-RPs, and NTLBG-RPs) which have been introduced in Section 3 to form four different populations by resampling. Specifically, *N* denotes the number of representative points used for each distribution, while *n* represents the sample size generated in each resampling procedure. We then employ the samples obtained via resampling for statistical inference. The steps of resampling used in this paper are as follows:

Step 1. Generate a random number *U*, i.e., U∼U(0, 1); the latter is the uniform distribution on (0, 1).

Step 2. Define a random variable *Y* by

(18) $$Y=\left\{\begin{array}{cc} b_{1}, & \;\mathrm{when}\;U<p_{1},\\ b_{2}, & \;\mathrm{when}\;p_{1}⩽U<p_{1}+p_{2},\\ \vdots & \vdots \\ b_{k}, & \;\mathrm{when}\;\sum_{i=1}^{k-1}p_{i}⩽U. \end{array}\right.$$

Step 3. Repeat the above two steps n times, and we have a sample of *Y*, $y_{1},\ldots ,y_{n}$. Calculate the given statistic *T*.

Step 4. Repeat the above three steps 1000 times, and we obtain a sample of *T*, $T_{1},\ldots ,T_{1000}$.

Step 5. Use the mean of a sample of *T* to infer the statistic of the population.

Now we apply the four RP methods for estimation of four statistics of X∼IG(1, 1): mean, variance, skewness, and kurtosis. Table 3, Table 4 and Table 5 show estimation biases for the above four statistics, where QMC-RPs, FH-RPs, PKM-RPs, and NTLBG-RPs are employed involving the following cases: sample size n=30,50,100, and representative points k=5,15,30.

Based on the results in the Table 3, Table 4 and Table 5, the results reveal distinct performance patterns among the four methods. NTLBG-RPs demonstrates superior accuracy in mean and variance estimation, achieving near-zero biases across all configurations. FH-RPs and PKM-RPs excel in higher-moment characterization, particularly for kurtosis and skewness estimation. QMC-RPs provides reasonable mean estimates but shows significant limitations in capturing higher-order moments. This performance dichotomy suggests a fundamental trade-off between central moment accuracy and tail behavior characterization. The optimal method selection should therefore align with specific application requirements, prioritizing either distribution center or tail properties.

## 5. MLE via Quantile Estimators of IG Distribution

Let $x=(x_{1},x_{2},\ldots ,x_{n})$ be a set of n i.i.d samples from IG distribution with pdf f(x;θ). When θ=(μ,λ), then the log-likelihood function is defined as

(19) $$l\left(\mu ,\lambda ;x\right)=\sum_{i=1}^{n}\log f\left(x_{i};\mu ,\lambda \right)=\frac{n}{2}\log\lambda -\frac{n}{2}\log\left(2\pi \right)-\frac{3}{2}\sum_{i=1}^{n}\log x_{i}-\frac{\lambda }{2\mu^{2}}\sum_{i=1}^{n}\frac{{\left(x_{i}-\mu \right)}^{2}}{x_{i}}.$$

When θ=(α,μ,λ), the pdf f(x;θ) is

(20) $$\begin{array}{c} f\left(x\right)={\left(\frac{\lambda }{2\pi {\left(x-\alpha \right)}^{3}}\right)}^{1/2}\exp\left(-\frac{\lambda {\left(x-\alpha -\mu \right)}^{2}}{2\mu^{2}\left(x-\alpha \right)}\right)I_{\left[\alpha ,\infty \right)}\left(x\right), \end{array}$$

and the log-likelihood function is defined as

(21) $$l\left(\alpha ,\mu ,\lambda ;x\right)=\frac{n}{2}\log\lambda -\frac{n}{2}\log\left(2\pi \right)-\frac{3}{2}\sum_{i=1}^{n}\log\left(x_{i}-\alpha \right)-\frac{\lambda }{2\mu^{2}}\sum_{i=1}^{n}\frac{{\left(x_{i}-\alpha -\mu \right)}^{2}}{x_{i}-\alpha }.$$

The goal of MLE is to find the model parameters θ that can maximize the log-likelihood function over the parameter space Θ, that is,

(22) $$\begin{array}{c} {\hat{\theta }}_{MLE}=\arg\max_{\theta \in \Theta }l\left(\theta ;x\right). \end{array}$$

The sequential number theoretic optimization algorithm (SNTO), introduced by Fang and Wang [27], represents a broadly applicable optimization technique. Subsequently, this SNTO algorithm can be employed to determine the numerical solutions ${\hat{\theta }}_{MLE}$ through the maximization of Equations (19) and (21).

**Figure 3:**
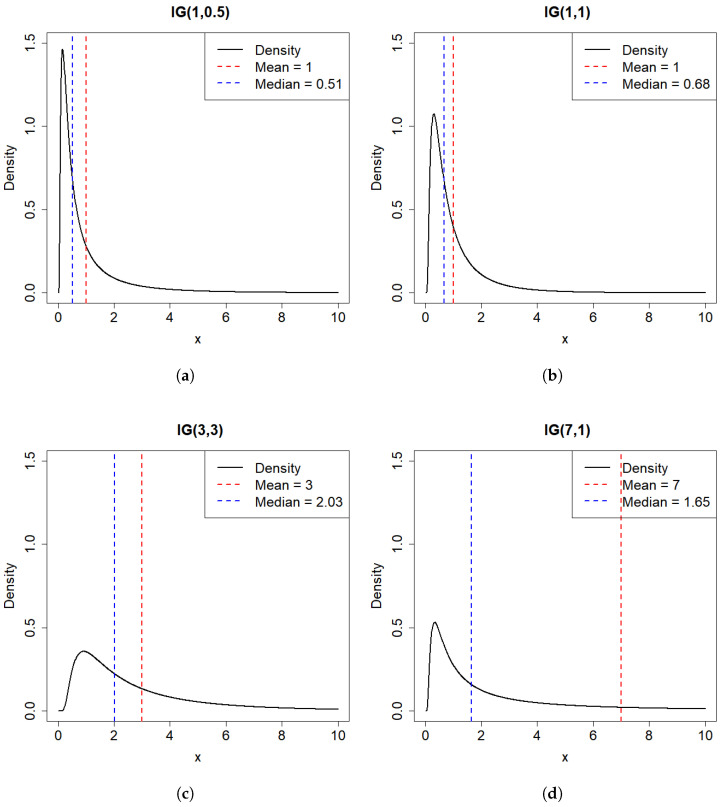
(**a**) Density plot of IG(1, 0.5). (**b**) Density plot of IG(1, 1). (**c**) Density plot of IG(7, 1). (**d**) Density plot of IG(3, 3).

In this part, we study parameter estimation of four two-parameter Inverse Gaussian distributions (IG(1,1), IG(1,0.5), IG(7,1), IG(3,3)) and one three-parameter Inverse Gaussian distribution IG(1,0.5,1). Some results for IG(1,0.5), IG(7,1), IG(3,3) are displayed in the Appendix A to save space. The density plots corresponding to these four two-parameter distributions are shown in Figure 3. The three-parameter inverse Gaussian distribution is derived from the two-parameter inverse Gaussian distribution by adding a location shift parameter, which does not alter the shape of the distribution. Therefore, it need not be presented separately.

### 5.1. Two Nonparametric Quantile Estimators

#### 5.1.1. HD Estimator

The Harrell–Davis quantile estimator [19] consists of a linear combination of the order statistics, admitting a jackknife variance. The Harrell–Davis quantile estimator offers a significant gain in efficiency, with emphasis on small sample results. Let $x=\{x_{1},\ldots ,x_{n}\}$ be a random sample of size *n* from an inverse Gaussian distribution. Denote $X_{\left(i\right)}$ as the *i*-th largest value in *x* and $F^{-1}\left(p\right)$ as the *p*-th population quantile. The quantile estimator based on the random sample is proposed to be Q(p)

(23) $$Q\left(p\right)=\sum_{i=1}^{n}W_{n,i}X_{\left(i\right)}.$$

where

(24) $$\begin{array}{cc} W_{n,i} & =\frac{1}{B((n+1)p,(n+1)(1-p))}\int_{(i-1)/n}^{i/n}y^{(n+1)p-1}{\left(1-y\right)}^{(n+1)(1-p)-1}dy\\ \; & =I_{i/n}\left[\left(n+1\right)p,\left(n+1\right)\left(1-p\right)\right]-I_{(i-1)/n}\left[\left(n+1\right)p,\left(n+1\right)\left(1-p\right)\right], \end{array}$$

and $I_{x}\left(a,b\right)$ denotes the incomplete beta function.

#### 5.1.2. SV Estimators

The SV estimators, as proposed by Sfakianakis and Verginis [20], offer alternative methods for quantile estimation with advantages in small sample sizes and extreme quantiles. Let $x=\{x_{1},\ldots ,x_{n}\}$ be a random sample of size *n* from an inverse Gaussian distribution. The *q*-th quantile of the population, Q(q), is one of the $S_{i}$. Define the random variables $\delta_{i}$ as

(25) $$\delta_{i}=\left\{\begin{array}{cc} 1, & X_{\left(i\right)}\leq Q\left(q\right),\\ 0, & X_{\left(i\right)}>Q\left(q\right), \end{array}\right.$$

where $\delta_{i}∼\mathrm{Bernoulli}\left(q\right)$. The Bernoulli distribution is a discrete distribution with two possible outcomes: 1 (success) with probability *q* and 0 (failure) with probability 1−q. In this context, *q* represents the probability of success for each trial. Then, their sum $N=\delta_{1}+\delta_{2}+\ldots +\delta_{n}$ has a binomial distribution with probability *q*, supposing $\delta_{i}$ are independent. So, $P(Q\left(q\right)\in S_{i})=P\left(N=i\right)=B\left(i;n,q\right)$, where i=0,1,…,n. Let the random variable $\Psi \left(q\right)=Q_{i}{\left(q\right)}^{\prime }$, where $Q_{i}{\left(q\right)}^{\prime }$ is a point estimator of Q(q) conditioned on the event $Q\left(q\right)\in S_{i}$, i=0,1,…,n. An estimator of Q(q) is obtained by calculating Q(q)=E(Ψ(q)).

Three different definitions of $Q_{i}{\left(q\right)}^{\prime }$ are examined to derive three distinct quantile estimators, denoted as $Q_{SV1}\left(q\right)$, $Q_{SV2}\left(q\right)$, and $Q_{SV3}\left(q\right)$. Table 6 shows construction formulas of three quantile estimators and gives assumption of $Q_{0}{\left(q\right)}^{\prime }$ or $Q_{n}{\left(q\right)}^{\prime }$ of these estimators.

Substitute the above equations into $\Psi \left(q\right)=Q_{i}{\left(q\right)}^{\prime }$ and find the expectation Q(q)=E(Ψ(q)). After simplification, the following three SV estimators are obtained:

(26) $$\begin{array}{cc} Q_{SV1}\left(q\right) & =\frac{2B(0;n,q)+B(1;n,q)}{2}X_{\left(1\right)}+\frac{B\left(0;n,q\right)X_{\left(2\right)}-B\left(0;n,q\right)X_{\left(3\right)}}{2}\\ \; & \;+\sum_{i=2}^{n-1}\left[\frac{B(i;n,q)+B(i-1;n,q)}{2}X_{\left(i\right)}\right]-\frac{B\left(n;n,q\right)X_{\left(n-2\right)}+B\left(n;n,q\right)X_{\left(n-1\right)}}{2}\\ \; & \;+\frac{2B(n;n,q)+B(n-1;n,q)}{2}X_{\left(n\right)}, \end{array}$$

(27) $$\begin{array}{c} Q_{SV2}\left(q\right)=\sum_{i=0}^{n-1}B\left(i;n,q\right)X_{\left(i+1\right)}+\left(2X_{\left(n\right)}-X_{\left(n-1\right)}\right)B\left(n;n,q\right), \end{array}$$

(28) $$\begin{array}{c} Q_{SV3}\left(q\right)=\sum_{i=1}^{n}B\left(i;n,q\right)X_{\left(i\right)}+\left(2X_{\left(1\right)}-X_{\left(2\right)}\right)B\left(0;n,q\right). \end{array}$$

These four quantile estimators to QMC-data that approximate the inverse Gaussian distribution are applied to compare their effects in revising data in next section.

### 5.2. Estimation Accuracy Measures

In this subsection, six methods are showed for parameter estimation. These methods include the following: (1) the Plain method, which is the traditional maximum likelihood estimation (MLE) based on random samples with SNTO optimization; (2) the HD method, which uses QMC data (HD-quantiles) combined with SNTO optimization; (3) the SV1, SV2, and SV3 methods, which are based on QMC data (SV-quantiles) with different SV quantile constructions, also using SNTO optimization; and (4) the MLE-analytic formulas method, a traditional MLE approach based on analytical formulas without SNTO optimization.

There are many metrics to assess estimation precision. The following four accuracy measures, labeled (a) through (d), are examined in this study. Across 100 Monte Carlo iterations, the averages of these accuracy measures (a)–(d) are compiled in tables titled “average accuracy measures” for assessment purposes.The true distribution is represented by $F_{\mathrm{true}}$ or $f_{\mathrm{true}}$, while the estimated distributions are denoted as $F_{\mathrm{est}}$ or $f_{\mathrm{est}}$.

(a) The L2-distance between the cumulative distribution function (c.d.f.) (*F*) of the underlying distribution and its estimated distribution is considered. The L2-Distance (L2.cdf) for comparing two c.d.fs is expressed as:

(29) $$\begin{array}{c} L_{2}\left(F_{\mathrm{true}},F_{\mathrm{est}}\right)={\left\{\int_{R^{d}}{\left(F_{\mathrm{true}}\left(x\right)-F_{\mathrm{est}}\left(x\right)\right)}^{2}dx\right\}}^{1/2}. \end{array}$$

(b) The L2-distance between the probability density function (*f*) of the underlying distribution and its estimated distribution is considered. The L2-Distance (L2.pdf) for comparing two density functions is given by

(30) $$\begin{array}{c} L_{2}\left(f_{\mathrm{true}},f_{\mathrm{est}}\right)={\left\{\int_{R^{d}}{\left(f_{\mathrm{true}}\left(x\right)-f_{\mathrm{est}}\left(x\right)\right)}^{2}dx\right\}}^{1/2}. \end{array}$$

(c) The Kullback–Leibler divergence (KL), also known as relative entropy, serves as an indicator of the disparity between two probability distributions. The KL divergence from $F_{\mathrm{est}}$ to $F_{\mathrm{true}}$ is formulated as

(31) $$\begin{array}{c} D_{\mathrm{KL}}\left(F_{\mathrm{true}}∥F_{\mathrm{est}}\right)=\int_{-\infty }^{\infty }f_{\mathrm{true}}\left(x\right)\ln\left(\frac{f_{\mathrm{true}}\left(x\right)}{f_{\mathrm{est}}\left(x\right)}\right)dx. \end{array}$$

(d) The absolute bias index (ABI) is employed to quantify the overall bias in parameter estimation. Let $\hat{\mu }$ and $\hat{\lambda }$ represent the estimated values of μ and λ for the inverse Gaussian distribution, where μ>0 and λ>0. The ABI is defined as

(32) $$\begin{array}{c} ABI=\frac{1}{2}\left[\frac{|\mu -\hat{\mu }|}{\mu }+\frac{|\lambda -\hat{\lambda }|}{\lambda }\right]. \end{array}$$

#### 5.2.1. MLE of IG(μ,λ)

Five MLE-based methods are compared based on the sample size of n=30,50,100. Table 7 presents the average accuracy measures corresponding to L2-distances, KL-divergence, and ABI. For each metric, the optimal performance across different distributions and sample sizes is emphasized in bold. This bold-highlighting convention is consistently applied in the remaining tables.

From the result of Table 7, we can know the comparative analysis reveals that the Plain method consistently excels in L2.cdf (bold in 9/12 cases for IG(1, 1), IG(1, 0.5), IG(7, 1), and IG(3, 3)) and ABI (7/12 bold values), demonstrating robustness for cumulative distribution accuracy, while the HD method dominates L2.pdf (bold in 9/12 cases) and KL divergence (7/12 bold values), indicating superior probability density estimation. QSV variants show mixed results with occasional competitiveness but generally underperform systematically. Performance improves with sample size (n=30→100), particularly for HD and Plain, where error metrics decrease monotonically. Notably, IG(3, 3) exhibits unique behavior with shared dominance between Plain and HD, while QSV methods struggle, highlighting distribution-dependent efficacy. Overall, HD proves optimal for density-focused tasks (L2.pdf/KL) across most inverse Gaussian parameterizations, whereas the Plain method suits cumulative metrics (L2.cdf/ABI), with QSV methods lacking consistent advantages.

To better interpret the results presented in Table 7, we study the frequency of different rankings for the five methods across various metrics. The ranking of these methods is determined based on 100 Monte Carlo simulations, where each simulation records the relative performance of the methods across the specified accuracy metrics. The tables present the frequency of each method achieving ranks 1 through 5, providing a comprehensive assessment of their effectiveness. This analysis aims to identify the most robust method for parameter estimation under varying sample sizes (n=30,50,100).

The Table 8 presents the ranking distribution of five sampling methods (Plain, HD, QSV1, QSV2, QSV3) across four accuracy measures (L2.cdf, L2.pdf, KL, ABI) based on 100 Monte Carlo simulations for sample sizes (n=30,50,100) of IG(1, 1). Since the results for IG(1, 0.5), IG(7, 1), and IG(3, 3) are similar to those for IG(1, 1), and to save space, they are not individually presented in the main text but are included in Appendix A, which readers may refer to for further details.

In Table 8, the Plain method consistently ranks third or lower across all measures and sample sizes, indicating stable but suboptimal performance. HD performs moderately, often ranking second or third, with occasional first-place rankings, suggesting reliability but limited excellence. QSV1 and QSV2 frequently achieve first-place rankings, particularly in KL and ABI for larger *n*, but also show higher variability, with notable fifth-place occurrences, indicating sensitivity to specific conditions. QSV3 exhibits balanced performance, with frequent first- and second-place rankings, especially in L2.pdf and ABI at (n=100), suggesting robustness across diverse measures. As *n* increases, QSV2 and QSV3 generally improve in top rankings, while Plain and HD remain consistent but less competitive.

The analysis of Table 9 and Table 10 (with additional results for IG(1, 0.5) and IG(7, 1) provided in Appendix A, Table A4 and Table A5) reveals distinct performance patterns across the inverse Gaussian distributions. A consistent pattern emerges for the IG(1, 1) and IG(7, 1) distributions (see Table 9 and Table A5), where the QSV3 method consistently outperforms other contenders in density-related metrics (L2.pdf and KL divergence), achieving the lowest errors across nearly all sample sizes. This makes QSV3 the recommended choice for density-focused tasks. For parameter estimation under these distributions, the Plain method provides the most accurate estimates of μ, especially at smaller sample sizes. However, a different dynamic is observed for the IG(3, 3) distribution (Table 10). Here, the dominance shifts, with Plain and HD methods sharing the lead. Notably, the HD method demonstrates exceptional performance at the largest sample size (n=100), achieving optimal values in the majority of metrics. This indicates that for distributions with certain parameter configurations, traditional discretization methods can be highly effective for parameter estimation at larger *n*.

Overall, the choice of optimal discretization method is context-dependent. We recommend QSV3 for applications prioritizing density estimation, Plain for cumulative distribution metrics and μ estimation at small *n*, and HD for parameter estimation tasks when dealing with larger samples from certain distributions. The Analytic formulas method occasionally excels in specific scenarios but lacks consistency, while QSV1 and QSV2 exhibit limited advantages in this study.

#### 5.2.2. MLE of IG(μ,α,λ)

In this subsection, parameter estimation for the three-parameter inverse Gaussian distribution IG(1,0.5,1) is presented. We know that obtaining an analytical MLE solution for the three-parameter inverse Gaussian distribution is challenging due to the multimodal nature of its log-likelihood function, particularly the complexity in estimating α, which renders direct solutions to the derivative equations impractical. To overcome the limitations of analytical solutions, numerical optimization methods such as SNTO can be used. We employed the SNTO algorithm on a random sample for parameter estimation in the optimization step, comparing its performance with the traditional MLE method for estimating $\hat{\mu }$ and $\hat{\lambda }$ based on analytical expressions at a given α.

Table 11 presents parameter estimation results for the IG(1, 0.5, 1)) distribution across five methods (Plain, HD, QSV1, QSV2, QSV3) and three sample sizes (n=30,50,100) across four accuracy measures (L2.pdf, L2.cdf, KL, ABI). QSV3 demonstrates superior performance at n=30 and n=50, achieving the lowest errors in L2.pdf, L2.cdf, and KL, indicating its effectiveness for smaller samples. At n=100, HD outperforms others with the lowest errors across all measures, suggesting its strength with larger samples. Plain shows consistent but moderate performance, often excelling in ABI, while QSV1 and QSV2 exhibit higher variability with suboptimal rankings across measures. The results highlight QSV3’s robustness at lower *n* and HD’s dominance as sample size increases.

## 6. Case Study

In this section, we utilize inverse Gaussian distribution to model a set of data, focusing on the engineering planning and design. The data set is taken from Example 7 by Chhikara and Folks (1978) [8].

The data set gives 25 runoff amounts at Jug Bridge, Maryland, as shown in Table 12.

**Figure 4:**
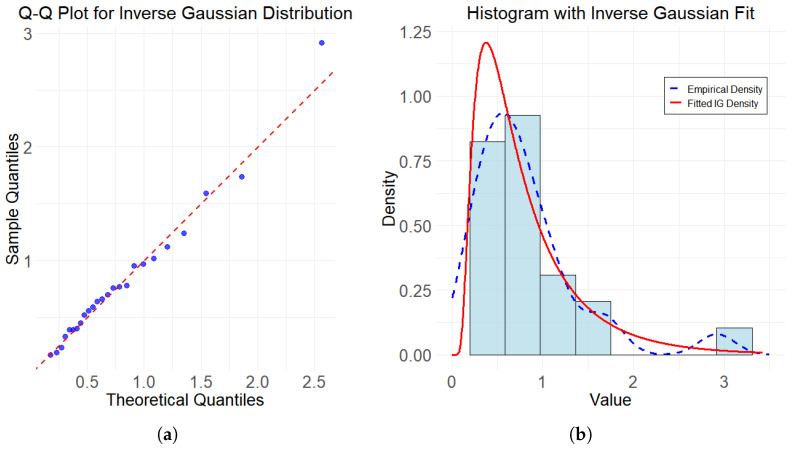
(**a**) Q-Q Plot for inverse Gaussian distribution. (**b**) Histogram with inverse Gaussian fit.

In the above case, the histogram and Q-Q plot of the data set are shown in Figure 4. According to fitted IG density, empirical density, and Q-Q plot, data set tends to inverse Gaussian distribution. Using the graphical method to determine Inverse Gaussian distribution has limitations. Therefore, it is also necessary to conduct a K-S test on the data to avoid misspecification of the data distribution type.

The K-S test statistic for Example is D=0.0621 with a *p*-value of 0.9976. Therefore, it is reasonable to assume that the data set follows inverse Gaussian distribution. We use the K-S test and three performance indicators to compare the fitting effects of these five methods, i.e., the bias, the sum of squares due to error (SSE), and the coeffcient of determination ($R^{2}$).

Table 13 presents the MLE parameter values obtained by different methods, and shows the parameter estimates, maximum likelihood function values, results of the K-S goodness of fit test, and performance indicators among several methods. For each measure, the methods that perform the best are marked in bold to facilitate easier comparison. Among the several revised methods considered, QSV1-MLE demonstrates the best fitting performance.

## 7. Conclusions

This paper investigates the statistical simulation and parameter estimation of the IG distribution, studying and comparing five RPs methods to enhance inference accuracy and employing QMC-data revisions with HD and SV quantile estimators for MLE. The paper demonstrates that the superiority of MSE-RPs in moment and density estimation, alongside their effectiveness in resampling accuracy, underscores their potential for improving inference precision. The integration of QMC-data revisions with HD and SV quantile estimators, particularly QSV1 and QSV3, optimizes MLE performance across various sample sizes and parameter scenarios, as validated by the real runoff dataset. These findings suggest that MSE-RPs, combined with tailored quantile approaches, provide a reliable framework for addressing the challenges of skewed distributions, with broad implications for fields such as lifetime analysis, satellite communications, and risk modeling, thereby advancing the accuracy of entropy-based evaluations in practical settings.

## Figures and Tables

**Table 1:** Estimation bias of variance, kurtosis, mean, and skewness.

Statistic	Category	5	10	15	20	25	28	30
Variance	MC-RPs (10)	−0.5105	−0.2431	−0.2357	−0.3721	−0.0064	−0.1115	0.2320
	QMC-RPs	−0.5423	−0.3695	−0.2883	−0.2396	−0.2067	−0.1914	−0.1826
	FH-RPs	**−0.0833**	**−0.0236**	**−0.0109**	**−0.0063**	**−0.0041**	**−0.0033**	**−0.0029**
	PKM-RPs	−0.0833	−0.0236	−0.0109	−0.0063	−0.0041	−0.0033	−0.0029
	NTLBG-RPs	−0.0846	−0.0262	−0.0163	−0.0132	−0.0115	−0.0108	−0.0104
Kurtosis	MC-RPs (10)	−15.5451	−13.8874	−13.0316	−13.1384	−11.6139	−12.1894	−11.7499
	QMC-RPs	−15.5670	−13.9531	−12.8774	−12.0745	−11.4366	−11.1093	−10.9094
	FH-RPs	**−4.8634**	**−1.9262**	**−1.0333**	**−0.6452**	**−0.4416**	**−0.3628**	**−0.3214**
	PKM-RPs	−4.8634	−1.9262	−1.0333	−0.6452	−0.4416	−0.3628	−0.3215
	NTLBG-RPs	−5.0921	−2.8455	−2.5595	−2.4208	−2.2783	−2.2534	−2.2494
Mean	MC-RPs (10)	−0.0208	−0.0246	0.0187	−0.0755	0.0115	0.0384	0.0555
	QMC-RPs	−0.0857	−0.0459	−0.0316	−0.0242	−0.0196	−0.0177	−0.0165
	FH-RPs	**0.0000**	**0.0000**	**0.0000**	**0.0000**	**0.0000**	**0.0000**	**0.0000**
	PKM-RPs	0.0000	0.0000	0.0000	0.0000	0.0000	0.0000	0.0000
	NTLBG-RPs	−0.0001	−0.0001	−0.0001	−0.0001	−0.0001	−0.0001	−0.0001
Skewness	MC-RPs (10)	−2.2837	−1.6462	−1.5081	−1.5186	−1.0662	−1.3430	−1.1255
	QMC-RPs	−2.0808	−1.6007	−1.3632	−1.2107	−1.1010	−1.0480	−1.0166
	FH-RPs	**−0.2515**	**−0.0844**	**−0.0420**	**−0.0251**	**−0.0166**	**−0.0135**	**−0.0119**
	PKM-RPs	−0.2515	−0.0844	−0.0420	−0.0251	−0.0166	−0.0135	−0.0119
	NTLBG-RPs	−0.2723	−0.1493	−0.1405	−0.1326	−0.1240	−0.1218	−0.1210

The minimum estimation bias within each statistic and sample size is highlighted in bold.

**Table 2:** Recommended *h* and $L_{2}$-distance under n = 30.

		QMC-RPs	MSE-RPs	PKM-RPs	NTLBG-RPs
Zone	zone 1	0–1	0–1	0–1	0–1
	zone 2	1–15	1–15	1–15	1–15
*h*	$h_{1}$	0.0500	0.0543	0.0543	0.0591
	$h_{2}$	0.2713	0.1417	0.1417	0.2418
$D_{2}^{2}$	$D_{12}^{2}$	0.03091647	0.03381734	0.03380385	0.05222610
	$D_{22}^{2}$	0.03368552	0.00687223	0.00687258	0.00966432

**Table 3:** Estimation bias for k=5 by resampling.

Statistic	Method	n=30	n=50	n=100
Variance	QMC-RPs	−0.5573	−0.5519	−0.5471
	FH-RPs	−0.7920	−0.7917	−0.7928
	PKM-RPs	−0.7937	−0.7922	−0.7932
	NTLBG-RPs	**−0.0550**	**−0.0697**	**−0.0796**
Kurtosis	QMC-RPs	−15.2659	−15.3839	−15.4725
	FH-RPs	1.3644	1.3715	1.6490
	PKM-RPs	**1.2462**	**1.3232**	**1.5490**
	NTLBG-RPs	−4.6785	−4.8899	−4.9691
Mean	QMC-RPs	−0.0873	−0.0867	−0.0870
	FH-RPs	−0.3691	−0.3714	−0.3724
	PKM-RPs	−0.3690	−0.3692	−0.3712
	NTLBG-RPs	**0.0336**	**0.0174**	**0.0077**
Skewness	QMC-RPs	−2.0433	−2.0550	−2.0660
	FH-RPs	0.1559	0.1836	0.2152
	PKM-RPs	**0.1443**	**0.1639**	**0.1993**
	NTLBG-RPs	−0.2644	−0.2695	−0.2708

The minimum estimation bias within each statistic and sample size is highlighted in bold.

**Table 4:** Estimation bias for k=15 by resampling.

Statistic	Method	n=30	n=50	n=100
Variance	QMC-RPs	−0.3188	−0.3134	−0.2980
	FH-RPs	−0.7672	−0.7664	−0.7672
	PKM-RPs	−0.7693	−0.7671	−0.7679
	NTLBG-RPs	**−0.0085**	**−0.0116**	**−0.0125**
Kurtosis	QMC-RPs	−12.9503	−12.6811	−12.7568
	FH-RPs	**−1.5896**	−1.5647	**−1.1952**
	PKM-RPs	−1.6255	**−1.4699**	−1.2064
	NTLBG-RPs	−1.7125	−2.0773	−2.3439
Mean	QMC-RPs	−0.0391	−0.0414	−0.0352
	FH-RPs	−0.3784	−0.3794	−0.3800
	PKM-RPs	−0.3773	−0.3770	−0.3784
	NTLBG-RPs	**0.0207**	**0.0128**	**0.0060**
Skewness	QMC-RPs	−1.4538	−1.3787	−1.3668
	FH-RPs	−0.2725	−0.2548	−0.2210
	PKM-RPs	−0.2858	−0.2661	−0.2320
	NTLBG-RPs	**−0.1076**	**−0.1224**	**−0.1335**

The minimum estimation bias within each statistic and sample size is highlighted in bold.

**Table 5:** Estimation bias for k=30 by resampling.

Statistic	Method	n=30	n=50	n=100
Variance	QMC-RPs	−0.2065	−0.1950	−0.1872
	FH-RPs	−0.7659	−0.7654	−0.7657
	PKM-RPs	−0.7672	−0.7655	−0.7658
	NTLBG-RPs	**−0.0070**	**−0.0051**	**−0.0060**
Kurtosis	QMC-RPs	−12.1054	−11.3387	−11.0403
	FH-RPs	−1.5183	**−1.5125**	**−1.2570**
	PKM-RPs	−1.6669	−1.5214	−1.3705
	NTLBG-RPs	**−1.2137**	−1.8096	−1.9828
Mean	QMC-RPs	−0.0204	−0.0210	−0.0180
	FH-RPs	−0.3739	−0.3745	−0.3760
	PKM-RPs	−0.3728	−0.3723	−0.3749
	NTLBG-RPs	**0.0052**	**0.0038**	**0.0006**
Skewness	QMC-RPs	−1.2871	−1.1344	−1.0679
	FH-RPs	−0.2768	−0.2631	−0.2361
	PKM-RPs	−0.2979	−0.2808	−0.2552
	NTLBG-RPs	**−0.1006**	**−0.1127**	**−0.1084**

The minimum estimation bias within each statistic and sample size is highlighted in bold.

**Table 6:** Definitions of three quantile estimators.

SV Estimators	Construction Formula	Assumptions
$Q_{SV1}\left(q\right)$	$Q_{i}{\left(q\right)}^{\prime }=\frac{X_{\left(i\right)}+X_{\left(i+1\right)}}{2},$	$Q_{0}{\left(q\right)}^{\prime }-Q_{1}{\left(q\right)}^{\prime }=Q_{1}{\left(q\right)}^{\prime }-Q_{2}{\left(q\right)}^{\prime }$,
	i=1,2,…,n−1	$Q_{n}{\left(q\right)}^{\prime }-Q_{n-1}{\left(q\right)}^{\prime }=Q_{n-1}{\left(q\right)}^{\prime }-Q_{n-2}{\left(q\right)}^{\prime }$
$Q_{SV2}\left(q\right)$	$Q_{i}{\left(q\right)}^{\prime }=\sup\left(Y\in S_{j}\right)=X_{\left(i+1\right)},$	$Q_{n}{\left(q\right)}^{\prime }-Q_{n-1}{\left(q\right)}^{\prime }=Q_{n-1}{\left(q\right)}^{\prime }-Q_{n-2}{\left(q\right)}^{\prime }$
	i=0,1,…,n−1	
$Q_{SV3}\left(q\right)$	$Q_{i}{\left(q\right)}^{\prime }=\inf\left(Y\in S_{j}\right)=X_{\left(i\right)},$	$Q_{0}{\left(q\right)}^{\prime }-Q_{1}{\left(q\right)}^{\prime }=Q_{1}{\left(q\right)}^{\prime }-Q_{2}{\left(q\right)}^{\prime }$
	i=1,2,…,n	

**Table 7:** MLE estimations—average accuracy measures (n=30,50,100;N=100).

Type	Method	n=30				n=50				n=100			
		L2.pdf	L2.cdf	KL	ABI	L2.pdf	L2.cdf	KL	ABI	L2.pdf	L2.cdf	KL	ABI
IG(1,1)	Plain	0.1335	**0.0805**	0.0423	**0.1927**	0.1001	**0.0611**	0.0208	**0.1402**	0.0683	**0.0411**	0.0104	0.0950
IG(1,1)	HD	**0.1333**	0.0841	**0.0421**	0.1997	**0.0984**	0.0614	**0.0201**	0.1408	**0.0680**	0.0419	**0.0101**	0.0966
IG(1,1)	QSV1	0.1422	0.0850	0.0555	0.2088	0.1059	0.0636	0.0268	0.1514	0.0715	0.0456	0.0126	0.1001
IG(1,1)	QSV2	0.1429	0.1037	0.0450	0.2295	0.1014	0.0702	0.0221	0.1553	0.0716	0.0507	0.0112	0.1064
IG(1,1)	QSV3	0.1522	0.0846	0.0478	0.1962	0.1176	0.0647	0.0241	0.1497	0.0697	0.0443	0.0105	**0.0946**
IG(1,0.5)	Plain	0.1607	**0.0887**	**0.0483**	**0.2396**	0.1119	**0.0624**	0.0232	**0.1596**	0.0761	**0.0463**	0.0099	0.1101
IG(1,0.5)	HD	**0.1604**	0.0919	0.0485	0.2540	**0.1104**	0.0642	**0.0217**	0.1647	**0.0751**	0.0466	**0.0095**	0.1114
IG(1,0.5)	QSV1	0.1757	0.0954	0.0692	0.2756	0.1174	0.0651	0.0283	0.1712	0.0781	0.0473	0.0115	0.1132
IG(1,0.5)	QSV2	0.1664	0.1031	0.0512	0.2849	0.1150	0.0788	0.0230	0.1943	0.0777	0.0537	0.0106	0.1259
IG(1,0.5)	QSV3	0.1865	0.0936	0.0529	0.2400	0.1427	0.0674	0.0284	0.1697	0.0783	0.0466	0.0098	**0.1090**
IG(7,1)	Plain	0.0799	**0.1010**	**0.0182**	**0.1525**	0.0577	0.0801	**0.0103**	**0.1176**	0.0379	0.0600	0.0049	0.0877
IG(7,1)	HD	**0.0788**	0.1023	0.0190	0.1579	0.0572	**0.0795**	0.0104	0.1184	**0.0375**	**0.0588**	**0.0049**	**0.0859**
IG(7,1)	QSV1	0.0789	0.1032	0.0201	0.1619	0.0585	0.0811	0.0112	0.1231	0.0378	0.0601	0.0053	0.0885
IG(7,1)	QSV2	0.0789	0.1046	0.0204	0.1632	**0.0567**	0.0797	0.0111	0.1220	0.0380	0.0597	0.0053	0.0886
IG(7,1)	QSV3	0.0964	0.1128	0.0232	0.1587	0.0760	0.0951	0.0148	0.1305	0.0400	0.0608	0.0049	0.0872
IG(3,3)	Plain	0.0463	**0.0946**	**0.0132**	0.1205	0.0446	0.0902	0.0116	0.1099	0.0349	**0.0710**	0.0078	**0.0838**
IG(3,3)	HD	0.0465	0.0977	0.0133	0.1240	0.0438	**0.0887**	0.0110	0.1098	**0.0348**	0.0722	**0.0075**	0.0858
IG(3,3)	QSV1	**0.0458**	0.0973	0.0141	**0.1203**	0.0440	0.0906	0.0123	**0.1093**	0.0354	0.0724	0.0087	0.0851
IG(3,3)	QSV2	0.0489	0.1080	0.0137	0.1317	**0.0428**	0.0920	**0.0105**	0.1133	0.0359	0.0781	0.0078	0.0919
IG(3,3)	QSV3	0.0491	0.0961	0.0136	0.1218	0.0491	0.0925	0.0123	0.1146	0.0362	0.0717	0.0081	0.0859

The best performance within each measure per distribution and sample size is highlighted in bold.

**Table 8:** The rank of 5 methods in 4 accuracy measures (IG(1, 1)).

*n*	Method	L2.cdf					L2.pdf				
		Rank1	Rank2	Rank3	Rank4	Rank5	Rank1	Rank2	Rank3	Rank4	Rank5
30	Plain	8	26	65	1	0	8	34	40	18	0
30	HD	13	35	13	38	1	5	36	44	15	0
30	QSV1	31	13	4	17	35	27	10	8	29	26
30	QSV2	29	3	3	7	58	25	11	5	17	42
30	QSV3	19	23	15	37	6	35	9	3	21	32
		KL					ABI				
30	Plain	6	35	50	9	0	9	40	37	13	1
30	HD	7	40	31	22	0	9	24	45	22	0
30	QSV1	31	2	9	11	47	32	8	5	15	40
30	QSV2	27	6	5	27	35	21	9	5	24	41
30	QSV3	29	17	5	31	18	29	19	8	26	18
		L2.cdf					L2.pdf				
50	Plain	5	20	72	3	0	8	36	29	27	0
50	HD	12	40	12	36	0	5	35	50	10	0
50	QSV1	26	21	7	10	36	21	12	12	34	21
50	QSV2	36	5	2	6	51	30	13	7	19	31
50	QSV3	21	14	7	45	13	36	4	2	10	48
		KL					ABI				
50	Plain	5	41	43	11	0	5	50	17	28	0
50	HD	8	44	29	19	0	10	25	44	21	0
50	QSV1	26	6	13	19	36	24	15	17	17	27
50	QSV2	32	7	4	18	39	26	5	12	19	38
50	QSV3	29	2	11	33	25	35	5	10	15	35
		L2.cdf					L2.pdf				
100	Plain	3	14	78	5	0	2	34	44	20	0
100	HD	5	44	11	40	0	2	44	33	21	0
100	QSV1	30	14	4	16	36	20	16	10	27	27
100	QSV2	40	1	2	5	52	33	5	3	20	39
100	QSV3	22	27	5	34	12	43	1	10	12	34
		KL					ABI				
100	Plain	1	31	49	19	0	6	43	15	36	0
100	HD	4	45	21	30	0	5	24	47	24	0
100	QSV1	32	8	4	14	42	27	20	10	12	31
100	QSV2	31	6	6	19	38	29	4	9	19	39
100	QSV3	32	10	20	18	20	33	9	19	9	30

**Table 9:** Estimation results on IG(1, 1), *N* = 100.

*n*	Method	L2.pdf	L2.cdf	KL	ABI	$\hat{\mu }$	$\hat{\lambda }$
30	Plain	0.04980	0.01671	0.00286	0.05779	**1.00509**	1.11049
30	HD	0.06393	0.02833	0.00440	0.08200	1.03902	1.12497
30	QSV1	0.08129	0.03122	0.01076	0.12560	0.95727	1.20847
30	QSV2	0.10074	0.06753	0.01303	0.14175	1.13144	1.15207
30	QSV3	**0.01142**	**0.01127**	**0.00043**	**0.02244**	0.97528	**1.02015**
30	Analytic formulas	0.05243	0.01728	0.00358	0.06668	0.99020	1.12355
50	Plain	0.02534	**0.00914**	0.00092	0.03520	0.98907	1.05946
50	HD	0.03076	0.01272	0.00098	0.03766	1.01584	1.05948
50	QSV1	0.05169	0.02467	0.00505	0.08667	0.95599	1.12933
50	QSV2	0.05874	0.04148	0.00454	0.07973	1.08267	1.07678
50	QSV3	**0.01877**	0.01447	**0.00052**	**0.02331**	0.97068	**0.98270**
50	Analytic formulas	0.04398	0.01474	0.00221	0.05065	**1.00447**	1.09683
100	Plain	0.01481	**0.00584**	0.00034	0.02172	**0.99134**	1.03478
100	HD	0.01450	0.00672	0.00021	0.01794	1.01011	1.02578
100	QSV1	0.02855	0.01487	0.00160	0.04883	0.97173	1.06940
100	QSV2	0.03223	0.02508	0.00152	0.04259	1.05160	1.03357
100	QSV3	0.01188	0.00900	**0.00020**	**0.01492**	0.98183	**0.98833**
100	Analytic formulas	**0.00624**	0.01302	0.00030	0.02123	0.98855	1.03101

The best performance within each measure is highlighted in bold.

**Table 10:** Estimation results on IG(3, 3), N=100.

n	Method	L2.pdf	L2.cdf	KL	ABI	$\hat{\mu }$	$\hat{\lambda }$
30	Plain	0.00711	**0.00734**	**0.00016**	**0.01392**	**3.00712**	3.07637
30	HD	0.01284	0.02280	0.00056	0.02875	3.07231	3.10020
30	QSV1	0.01683	0.02596	0.00166	0.04962	2.91507	3.21281
30	QSV2	0.02414	0.05617	0.00254	0.05589	3.20040	3.13492
30	QSV3	**0.00643**	0.01504	0.00019	0.01394	2.94697	**2.96938**
30	Analytic formulas	0.03027	0.02994	0.00358	0.06668	2.97059	3.37065
50	Plain	**0.00519**	**0.00796**	**0.00015**	**0.01506**	2.97368	3.06402
50	HD	0.00777	0.01213	0.00019	0.01698	3.03533	**3.06654**
50	QSV1	0.01484	0.02948	0.00159	0.04801	2.89459	3.18262
50	QSV2	0.01959	0.04771	0.00178	0.04483	3.17242	3.09655
50	QSV3	0.01123	0.02132	0.00044	0.02437	2.93001	2.92375
50	Analytic formulas	0.02539	0.02553	0.00221	0.05065	**3.01341**	3.29050
100	Plain	0.00492	0.00875	0.00015	0.01519	2.96921	3.06037
100	HD	**0.00469**	**0.00720**	**0.00007**	**0.01018**	**3.02073**	**3.04032**
100	QSV1	0.01117	0.02341	0.00094	0.03670	2.91492	3.13509
100	QSV2	0.01357	0.03421	0.00090	0.03048	3.12448	3.05841
100	QSV3	0.00817	0.01695	0.00026	0.01784	2.94240	2.95058
100	Analytic formulas	0.00752	0.01082	0.00030	0.02123	2.96566	3.09304

The best performance within each measure is highlighted in bold.

**Table 11:** Estimation results on IG(1, 0.5, 1), N=100.

n	Method	L2.pdf	L2.cdf	KL	ABI	$\hat{\mu }$	$\hat{\alpha }$	$\hat{\lambda }$
30	Plain	0.06271	0.01494	0.00928	**0.02120**	0.98465	0.52369	1.00088
30	HD	0.07647	0.02574	0.01829	0.03954	1.01264	0.53394	0.96188
30	QSV1	0.05848	0.06164	0.00823	0.05831	1.15003	0.49167	1.00825
30	QSV2	0.12524	0.04358	0.04314	0.05009	1.02293	0.53946	1.04841
30	QSV3	**0.01719**	**0.01117**	**0.00073**	0.03444	0.98345	0.49372	1.07421
50	Plain	0.04974	0.01349	0.00482	**0.01919**	0.99446	0.51553	1.02097
50	HD	0.05489	0.02092	0.00781	0.02797	1.01616	0.52314	0.97853
50	QSV1	0.03694	0.04825	0.00538	0.05698	1.11840	0.48912	1.03078
50	QSV2	0.08767	0.03141	0.01780	0.03404	1.02009	0.52820	1.02574
50	QSV3	**0.02644**	**0.00981**	**0.00084**	0.03741	0.99845	0.49272	1.09612
100	Plain	0.04974	0.00804	0.00482	0.01876	0.98980	0.50272	1.04063
100	HD	**0.00727**	**0.00777**	0.00095	**0.00687**	1.00415	0.50791	1.00065
100	QSV1	0.03694	0.02574	0.00240	0.05140	1.06915	0.48445	1.05395
100	QSV2	0.04340	0.01254	0.00350	0.01594	0.99890	0.51301	1.02071
100	QSV3	0.01859	0.00846	**0.00091**	0.04493	0.99796	0.48500	1.10276

The best performance within each measure is highlighted in bold.

**Table 12:** Data for example.

0.17	0.19	0.23	0.33	0.39
0.39	0.40	0.45	0.52	0.56
0.59	0.64	0.66	0.70	0.76
0.77	0.78	0.95	0.97	1.02
1.12	1.24	1.59	1.74	2.92

**Table 13:** The estimation result of real data.

Method	$\hat{\mu }$	$\hat{\lambda }$	lnL	K-S	*p*-Value	Bias	SSE	R^2^
Plain	0.8032	1.4397	**−14.3916**	0.0621	0.9976	0.0134	0.0261	0.9875
HD	0.8305	1.4418	−14.4158	0.078	0.9981	0.0247	0.036	0.9828
QSV1	0.7691	1.6801	−14.5999	0.0733	**0.9993**	**0.0097**	**0.0214**	**0.9897**
QSV2	0.928	1.4146	−14.7918	0.126	0.8225	0.0582	0.1134	0.9476
QSV3	0.7762	1.3708	−14.4322	**0.0584**	0.9688	−0.002	0.0272	0.9869

The best performance within each measure is highlighted in bold.

## Data Availability

The data used in the case study were obtained from Reference [8]. Further details about the data sources can be found in the cited references. No new data were created in this study.

## Appendix A

**Table A1:** The rank of 5 methods in 4 accuracy measures (IG(1, 0.5)).

*n*	Method	L2.cdf					L2.pdf				
		Rank1	Rank2	Rank3	Rank4	Rank5	Rank1	Rank2	Rank3	Rank4	Rank5
30	Plain	5	30	62	3	0	12	44	12	31	1
30	HD	9	39	12	40	0	4	24	67	5	0
30	QSV1	13	15	17	22	33	16	12	9	35	28
30	QSV2	39	5	1	7	48	26	11	9	24	30
30	QSV3	34	11	8	28	19	42	9	3	5	41
		KL					ABI				
30	Plain	9	42	36	13	0	15	44	16	25	0
30	HD	2	36	44	16	2	6	25	38	28	3
30	QSV1	18	5	6	29	42	18	5	28	15	34
30	QSV2	33	7	5	25	30	23	8	5	23	41
30	QSV3	38	10	9	17	26	38	18	13	9	22
		L2.cdf					L2.pdf				
50	Plain	10	30	53	7	0	11	40	12	37	0
50	HD	13	38	12	36	1	5	25	68	2	0
50	QSV1	21	19	20	19	21	15	16	16	41	12
50	QSV2	31	1	3	9	56	31	13	3	16	37
50	QSV3	25	12	12	29	22	38	6	1	4	51
		KL					ABI				
50	Plain	10	44	32	13	1	8	51	17	23	1
50	HD	9	39	37	14	1	10	28	29	32	1
50	QSV1	24	6	12	31	27	26	4	32	17	21
50	QSV2	26	5	7	21	41	25	3	4	17	51
50	QSV3	31	6	12	21	30	31	14	18	11	26
		L2.cdf					L2.pdf				
100	Plain	2	21	73	4	0	2	32	32	34	0
100	HD	10	42	4	44	0	3	38	48	11	0
100	QSV1	25	14	13	15	33	14	20	17	32	17
100	QSV2	38	5	1	6	50	35	7	1	19	38
100	QSV3	25	18	9	31	17	46	3	2	4	45
		KL					ABI				
100	Plain	1	31	47	21	0	5	37	24	34	0
100	HD	7	43	26	24	0	12	34	19	35	0
100	QSV1	24	13	7	25	31	18	15	33	11	23
100	QSV2	36	5	4	11	44	29	7	7	5	52
100	QSV3	32	8	16	19	25	36	7	17	15	25

**Table A2:** The rank of 5 methods in 4 accuracy measures (IG(7, 1)).

*n*	Method	L2.cdf					L2.pdf				
		Rank1	Rank2	Rank3	Rank4	Rank5	Rank1	Rank2	Rank3	Rank4	Rank5
30	Plain	18	33	8	37	4	19	33	6	36	6
30	HD	8	18	62	12	0	5	21	65	6	3
30	QSV1	13	29	17	29	12	15	29	12	30	14
30	QSV2	29	6	10	15	40	29	6	12	19	34
30	QSV3	42	10	6	5	37	41	11	5	5	38
		KL					ABI				
30	Plain	18	34	6	36	6	18	32	9	37	4
30	HD	8	18	64	6	4	9	17	60	14	0
30	QSV1	13	31	10	29	17	14	31	10	27	18
30	QSV2	30	7	14	19	30	28	9	17	13	33
30	QSV3	39	12	4	8	37	39	10	6	7	38
		L2.cdf					L2.pdf				
50	Plain	13	36	5	44	2	13	37	3	45	2
50	HD	5	18	66	11	0	3	17	75	5	0
50	QSV1	13	27	21	22	17	12	32	9	30	17
50	QSV2	37	10	5	20	28	38	8	6	19	29
50	QSV3	32	10	4	3	51	34	9	5	1	51
		KL					ABI				
50	Plain	14	34	4	45	3	13	35	9	40	3
50	HD	4	19	74	3	0	7	20	63	9	1
50	QSV1	13	29	10	29	19	16	22	16	27	19
50	QSV2	36	12	5	20	27	32	14	8	16	30
50	QSV3	33	8	6	3	50	32	9	7	6	46
		L2.cdf					L2.pdf				
100	Plain	2	37	19	42	0	4	42	11	43	0
100	HD	9	17	59	14	1	4	12	76	8	0
100	QSV1	13	30	10	29	18	22	28	4	31	15
100	QSV2	35	9	9	15	32	30	15	6	14	35
100	QSV3	41	8	2	1	48	40	4	2	5	49
		KL					ABI				
100	Plain	3	42	18	37	0	3	35	26	36	0
100	HD	3	20	68	9	0	7	25	52	12	4
100	QSV1	21	29	2	27	21	23	22	7	25	23
100	QSV2	34	7	7	24	28	31	12	7	23	27
100	QSV3	39	3	4	4	50	36	7	7	5	45

**Table A3:** The rank of 5 methods in 4 accuracy measures (IG(3, 3)).

*n*	Method	L2.cdf					L2.pdf				
		Rank1	Rank2	Rank3	Rank4	Rank5	Rank1	Rank2	Rank3	Rank4	Rank5
30	Plain	16	19	54	11	0	14	25	38	22	1
30	HD	13	27	21	35	4	12	29	31	22	6
30	QSV1	30	20	10	15	25	32	19	10	18	21
30	QSV2	30	7	4	12	47	22	19	12	12	35
30	QSV3	15	35	15	26	9	20	22	11	23	24
		KL					ABI				
30	Plain	13	25	47	14	1	13	27	41	18	1
30	HD	14	29	21	30	6	13	26	29	24	8
30	QSV1	31	16	15	10	28	34	16	10	13	27
30	QSV2	26	9	8	19	38	22	11	9	19	39
30	QSV3	20	30	10	29	11	21	33	11	23	12
		L2.cdf					L2.pdf				
50	Plain	6	13	72	9	0	11	19	43	27	0
50	HD	18	34	10	36	2	10	35	34	19	2
50	QSV1	32	16	9	13	30	27	18	14	23	18
50	QSV2	35	9	4	5	47	36	15	6	11	32
50	QSV3	13	27	9	35	16	20	12	7	18	43
		KL					ABI				
50	Plain	7	28	53	12	0	9	25	40	25	1
50	HD	11	40	17	29	3	13	25	29	31	2
50	QSV1	29	12	10	18	31	31	16	11	13	29
50	QSV2	37	7	8	11	37	30	13	11	10	36
50	QSV3	20	12	14	30	24	22	19	12	19	28
		L2.cdf					L2.pdf				
100	Plain	3	18	75	3	1	3	31	49	16	1
100	HD	9	40	7	43	1	5	37	28	29	1
100	QSV1	33	11	6	15	35	23	21	8	25	23
100	QSV2	40	2	3	4	51	36	4	5	10	45
100	QSV3	15	31	8	34	12	33	9	9	19	30
		KL					ABI				
100	Plain	2	29	52	17	0	8	36	28	28	0
100	HD	7	39	20	34	0	7	23	40	30	0
100	QSV1	34	10	2	13	41	31	21	7	9	32
100	QSV2	33	6	7	13	41	30	7	10	13	40
100	QSV3	24	18	18	22	18	24	16	14	19	27

**Table A4:** Estimation results on IG(1, 0.5), N=100.

n	Method	L2.pdf	L2.cdf	KL	ABI	$\hat{\mu }$	$\hat{\lambda }$
30	Plain	0.07335	0.02055	0.00462	0.07528	**0.99117**	0.57086
30	HD	0.09692	0.03336	0.00737	0.10874	1.04214	0.58768
30	QSV1	0.12600	0.03741	0.01631	0.15858	0.95335	0.63526
30	QSV2	0.13636	0.06959	0.01630	0.18828	1.15247	0.61204
30	QSV3	**0.02076**	**0.01404**	**0.00086**	**0.04045**	0.96127	**0.52109**
30	Analytic formulas	0.06303	0.01826	0.00368	0.07274	0.97843	0.56195
50	Plain	0.03534	**0.00983**	0.00105	0.03612	**0.99346**	0.53285
50	HD	0.04659	0.01997	0.00164	0.05583	1.03875	0.53646
50	QSV1	0.06421	0.02064	0.00429	0.08323	0.96340	0.56493
50	QSV2	0.07791	0.05360	0.00632	0.11825	1.13863	0.54893
50	QSV3	**0.01822**	0.01106	**0.00030**	**0.02424**	0.97320	**0.48916**
50	Analytic formulas	0.05150	0.01489	0.00242	0.05874	0.98228	0.54989
100	Plain	0.01899	**0.00528**	0.00030	0.01933	**0.99604**	0.51735
100	HD	0.02247	0.01172	0.00041	0.02889	1.02662	0.51558
100	QSV1	0.03241	0.01169	0.00116	0.04467	0.97479	0.53207
100	QSV2	0.04274	0.03316	0.00220	0.06649	1.08829	0.52234
100	QSV3	**0.00897**	0.00572	**0.00008**	**0.01226**	0.98588	**0.49481**
100	Analytic formulas	0.01556	0.00627	0.00029	0.02278	0.98500	0.51527

The best performance within each measure is highlighted in bold.

**Table A5:** Estimation results on IG(7, 1), N=100.

n	Method	L2.pdf	L2.cdf	KL	ABI	$\hat{\mu }$	$\hat{\lambda }$
30	Plain	0.02825	0.03129	0.00166	0.04631	6.93597	1.08347
30	HD	0.04269	0.04957	0.00380	0.06611	**7.02712**	1.12835
30	QSV1	0.05288	0.06005	0.00610	0.08786	6.92041	1.16435
30	QSV2	0.05545	0.06837	0.00651	0.09580	7.15371	1.16965
30	QSV3	**0.00079**	**0.00834**	**0.00002**	**0.00855**	6.89497	**1.00209**
30	Analytic formulas	0.04011	0.04516	0.00359	0.07640	6.79364	1.12331
50	Plain	0.01586	0.01745	0.00052	0.02738	6.93861	1.04598
50	HD	0.02339	0.02638	0.00110	0.03413	**6.99633**	1.06773
50	QSV1	0.03187	0.03539	0.00213	0.05279	6.92572	1.09496
50	QSV2	0.03388	0.04260	0.00230	0.05917	7.14069	1.09824
50	QSV3	**0.01103**	**0.01484**	**0.00022**	**0.02016**	6.91901	**0.97126**
50	Analytic formulas	0.03340	0.03711	0.00235	0.05578	6.91821	1.09988
100	Plain	0.00949	**0.01163**	0.00020	0.02062	6.90626	1.02784
100	HD	0.01157	0.01287	0.00026	0.01685	**6.99344**	1.03277
100	QSV1	0.01740	0.01949	0.00065	0.03298	6.89592	1.05109
100	QSV2	0.01859	0.02741	0.00067	0.03783	7.17362	1.05086
100	QSV3	**0.00579**	0.01180	**0.00007**	**0.01496**	6.89049	**0.98572**
100	Analytic formulas	0.01116	0.01402	0.00235	0.01919	7.05329	1.03077

The best performance within each measure is highlighted in bold.

**Table A6:** The points of RPs from IG(1, 1).

	category	RP1	RP2	RP3	RP4	RP5	RP6	RP7	RP8
n=5	QMC-RPs	0.237625	0.429742	0.675841	1.085120	2.143034			
	FH-RPs	0.412524	1.077368	2.061354	3.616510	6.523829			
	PKM-RPs	0.412523	1.077366	2.061348	3.616501	6.523817			
	NTLBG-RPs	0.410067	1.066664	2.035673	3.564004	6.421223			
n=10	QMC-RPs	0.184113	0.285325	0.379723	0.483171	0.604832	0.756110	0.956109	1.244060
	FH-RPs	0.282780	0.583336	0.940531	1.377229	1.918490	2.601214	3.486682	4.689563
	PKM-RPs	0.282778	0.583331	0.940523	1.377216	1.918472	2.601190	3.486653	4.689529
	NTLBG-RPs	0.261260	0.518738	0.820360	1.189870	1.653057	2.248230	3.034748	4.122999
n=15	QMC-RPs	0.162420	0.237625	0.300876	0.363621	0.429742	0.501927	0.582866	0.675841
	FH-RPs	0.230289	0.428358	0.643997	0.888024	1.167374	1.489236	1.862458	2.298770
	PKM-RPs	0.230287	0.428352	0.643986	0.888008	1.167351	1.489207	1.862421	2.298726
	NTLBG-RPs	0.177701	0.297268	0.420456	0.558135	0.718321	0.909491	1.142247	1.429964
n=20	QMC-RPs	0.149804	0.212175	0.261786	0.308643	0.355654	0.404373	0.455963	0.511506
	FH-RPs	0.200694	0.350971	0.506274	0.674670	0.859939	1.065068	1.293080	1.547407
	PKM-RPs	0.200685	0.350957	0.506254	0.674643	0.859904	1.065025	1.293027	1.547344
	NTLBG-RPs	0.137958	0.209947	0.277620	0.347437	0.422782	0.506585	0.601923	0.712768
n=25	QMC-RPs	0.141253	0.195791	0.237625	0.275954	0.313307	0.350897	0.389506	0.429742
	FH-RPs	0.181246	0.303859	0.426099	0.554903	0.693050	0.842349	1.004352	1.180611
	PKM-RPs	0.181242	0.303849	0.426083	0.554879	0.693018	0.842308	1.004300	1.180549
	NTLBG-RPs	0.120763	0.175902	0.224958	0.272780	0.321183	0.371487	0.424970	0.482953
n=28	QMC-RPs	0.137262	0.188371	0.226930	0.261786	0.295327	0.328667	0.362480	0.397263
	FH-RPs	0.172376	0.283337	0.392099	0.505183	0.625066	0.753237	0.890873	1.039072
	PKM-RPs	0.172371	0.283326	0.392081	0.505156	0.625031	0.753192	0.890818	1.039005
	NTLBG-RPs	0.115454	0.165381	0.208911	0.250482	0.291565	0.333181	0.376107	0.421139
n=30	QMC-RPs	0.134937	0.184113	0.220862	0.253827	0.285325	0.316417	0.347735	0.379723
	FH-RPs	0.167278	0.271810	0.373259	0.477921	0.588133	0.705240	0.830258	0.964093
	PKM-RPs	0.167272	0.271798	0.373239	0.477893	0.588096	0.705193	0.830200	0.964023
	NTLBG-RPs	0.112544	0.160032	0.200761	0.239180	0.277045	0.314999	0.353415	0.393126
	category	RP9	RP10	RP11	RP12	RP13	RP14	RP15	RP16
n=10	QMC-RPs	1.725360	2.922076						
	FH-RPs	6.466201	9.623196						
	PKM-RPs	6.466161	9.623159						
	NTLBG-RPs	5.750920	8.653409						
n=15	QMC-RPs	0.785355	0.918136	1.085120	1.305995	1.621860	2.143034	3.411364	
	FH-RPs	2.814540	3.433731	4.193535	5.156277	6.438620	8.301048	11.562225	
	PKM-RPs	2.814488	3.433673	4.193470	5.156207	6.438548	8.300979	11.562190	
	NTLBG-RPs	1.790194	2.248018	2.841775	3.632491	4.711291	6.288362	9.129651	
n=20	QMC-RPs	0.572167	0.639315	0.714672	0.800508	0.899950	1.017518	1.160114	1.339036
	FH-RPs	1.832177	2.152538	2.515086	2.928504	3.404570	3.959822	4.618516	5.418287
	PKM-RPs	1.832103	2.152453	2.514989	2.928395	3.404449	3.959688	4.618370	5.418128
	NTLBG-RPs	0.844104	1.001872	1.193483	1.429018	1.721482	2.089041	2.555589	3.153091
n=25	QMC-RPs	0.472161	0.517322	0.565834	0.618395	0.675841	0.739201	0.809779	0.889279
	FH-RPs	1.372816	1.582888	1.813084	2.066105	2.345241	2.654576	2.999263	3.385945
	PKM-RPs	1.372742	1.582803	1.812987	2.065994	2.345118	2.654439	2.999113	3.385782
	NTLBG-RPs	0.547016	0.618788	0.700777	0.795894	0.907606	1.040801	1.201331	1.396686
n=28	QMC-RPs	0.433433	0.471381	0.511506	0.554238	0.600059	0.649532	0.703326	0.762262
	FH-RPs	1.198965	1.371784	1.558920	1.761974	1.982824	2.223707	2.487319	2.776958
	PKM-RPs	1.198887	1.371694	1.558816	1.761856	1.982693	2.223561	2.487158	2.776784
	NTLBG-RPs	0.469147	0.521023	0.577773	0.640895	0.712346	0.794621	0.890395	1.003197
n=30	QMC-RPs	0.412743	0.447120	0.483171	0.521227	0.561647	0.604832	0.651251	0.701455
	FH-RPs	1.107649	1.261883	1.427848	1.606732	1.799900	2.008942	2.235732	2.482506
	PKM-RPs	1.107567	1.261788	1.427739	1.606609	1.799763	2.008790	2.235565	2.482323
	NTLBG-RPs	0.434701	0.478539	0.525587	0.576829	0.633202	0.696344	0.768272	0.850992
	category	RP17	RP18	RP19	RP20	RP21	RP22	RP23	RP24
n=20	QMC-RPs	1.574661	1.909444	2.456955	3.771838				
	FH-RPs	6.422164	7.748047	9.658992	12.981382				
	PKM-RPs	6.421995	7.747864	9.658797	12.981110				
	NTLBG-RPs	3.934887	4.993269	6.539831	9.328942				
n=25	QMC-RPs	0.979995	1.085120	1.209288	1.359587	1.547621	1.794286	2.143034	2.709786
	FH-RPs	3.823382	4.323461	4.902871	5.586080	6.411034	7.441252	8.795492	10.738640
	PKM-RPs	3.823206	4.323273	4.902671	5.585868	6.410811	7.441019	8.795250	10.738390
	NTLBG-RPs	1.636442	1.932897	2.303347	2.772290	3.376042	4.167427	5.223196	6.759912
n=28	QMC-RPs	0.827366	0.899950	0.981735	1.075041	1.183105	1.310626	1.464787	1.657343
	FH-RPs	3.096724	3.451793	3.848838	4.296661	4.807203	5.397224	6.091291	6.927496
	PKM-RPs	3.096535	3.451589	3.848621	4.296431	4.806960	5.396969	6.091024	6.927220
	NTLBG-RPs	1.138059	1.300997	1.498690	1.740975	2.041688	2.416649	2.887119	3.486758
n=30	QMC-RPs	0.756110	0.816036	0.882262	0.956109	1.039311	1.134207	1.244060	1.373608
	FH-RPs	2.751966	3.047418	3.372972	3.733821	4.136655	4.590302	5.106733	5.702749
	PKM-RPs	2.751767	3.047204	3.372743	3.733576	4.136396	4.590028	5.106445	5.702449
	NTLBG-RPs	0.947489	1.061775	1.198136	1.362230	1.561387	1.805856	2.107466	2.482508
	category	RP25	RP26	RP27	RP28	RP29	RP30	RP31	RP32
n=25	QMC-RPs	4.058467							
	FH-RPs	14.102428							
	PKM-RPs	14.102179							
	NTLBG-RPs	9.558364							
n=28	QMC-RPs	1.909444	2.265027	2.841156	4.206263				
	FH-RPs	7.969590	9.336769	11.294769	14.677905				
	PKM-RPs	7.969308	9.336459	11.294434	14.677578				
	NTLBG-RPs	4.267639	5.324128	6.847529	9.558364				
n=30	QMC-RPs	1.530093	1.725360	1.980711	2.340368	2.922076	4.296947		
	FH-RPs	6.402978	7.245596	8.294494	9.669086	11.635645	15.030035		
	PKM-RPs	6.402665	7.245272	8.294160	9.668744	11.635300	15.029713		
	NTLBG-RPs	2.954570	3.555009	4.328302	5.355569	6.847529	9.558364		

**Table A7:** The corresponding probabilities of RPs from IG(1, 1).

	category	P1	P2	P3	P4	P5	P6	P7	P8
n=5	QMC-RPs	0.200000	0.200000	0.200000	0.200000	0.200000			
	FH-RPs	0.543429	0.280605	0.122293	0.044281	0.009393			
	PKM-RPs	0.543427	0.280605	0.122294	0.044281	0.009393			
	NTLBG-RPs	0.539400	0.281200	0.123800	0.045600	0.010000			
n=10	QMC-RPs	0.100000	0.100000	0.100000	0.100000	0.100000	0.100000	0.100000	0.100000
	FH-RPs	0.303210	0.250172	0.171229	0.113110	0.072568	0.044597	0.025567	0.013023
	PKM-RPs	0.303207	0.250171	0.171229	0.113111	0.072568	0.044598	0.025567	0.013024
	NTLBG-RPs	0.259600	0.235000	0.174400	0.124400	0.085800	0.056800	0.034600	0.019000
n=15	QMC-RPs	0.066667	0.066667	0.066667	0.066667	0.066667	0.066667	0.066667	0.066667
	FH-RPs	0.197129	0.198779	0.159822	0.123196	0.093408	0.069955	0.051655	0.037432
	PKM-RPs	0.197125	0.198777	0.159821	0.123197	0.093409	0.069956	0.051656	0.037433
	NTLBG-RPs	0.099000	0.127800	0.126600	0.118600	0.108000	0.096200	0.083600	0.070200
n=20	QMC-RPs	0.050000	0.050000	0.050000	0.050000	0.050000	0.050000	0.050000	0.050000
	FH-RPs	0.139870	0.159043	0.140258	0.117302	0.096240	0.078195	0.063079	0.050521
	PKM-RPs	0.139858	0.159039	0.140256	0.117302	0.096241	0.078196	0.063081	0.050523
	NTLBG-RPs	0.041600	0.064000	0.072600	0.076000	0.077400	0.077400	0.076600	0.075200
n=25	QMC-RPs	0.040000	0.040000	0.040000	0.040000	0.040000	0.040000	0.040000	0.040000
	FH-RPs	0.105020	0.129728	0.121821	0.107483	0.092647	0.078981	0.066896	0.056387
	PKM-RPs	0.105013	0.129722	0.121816	0.107481	0.092646	0.078981	0.066897	0.056389
	NTLBG-RPs	0.024000	0.040000	0.047200	0.051000	0.052400	0.053600	0.054000	0.054800
n=28	QMC-RPs	0.035714	0.035714	0.035714	0.035714	0.035714	0.035714	0.035714	0.035714
	FH-RPs	0.090203	0.115859	0.112045	0.101330	0.089310	0.077762	0.067244	0.057882
	PKM-RPs	0.090195	0.115852	0.112040	0.101326	0.089309	0.077761	0.067245	0.057884
	NTLBG-RPs	0.019600	0.032800	0.039600	0.042600	0.044200	0.044800	0.045200	0.045400
n=30	QMC-RPs	0.033333	0.033333	0.033333	0.033333	0.033333	0.033333	0.033333	0.033333
	FH-RPs	0.082054	0.107826	0.106082	0.097321	0.086883	0.076561	0.066981	0.058328
	PKM-RPs	0.082046	0.107817	0.106076	0.097317	0.086881	0.076561	0.066981	0.058329
	NTLBG-RPs	0.017400	0.029600	0.035400	0.038600	0.040400	0.040800	0.040800	0.041200
	category	P9	P10	P11	P12	P13	P14	P15	P16
n=10	QMC-RPs	0.100000	0.100000						
	FH-RPs	0.005301	0.001224						
	PKM-RPs	0.005301	0.001224						
	NTLBG-RPs	0.008200	0.002200						
n=15	QMC-RPs	0.066667	0.066667	0.066667	0.066667	0.066667	0.066667	0.066667	
	FH-RPs	0.026432	0.018001	0.011639	0.006965	0.003678	0.001544	0.000366	
	PKM-RPs	0.026433	0.018002	0.011640	0.006965	0.003679	0.001544	0.000366	
	NTLBG-RPs	0.056600	0.043400	0.031200	0.020400	0.011400	0.005400	0.001600	
n=20	QMC-RPs	0.050000	0.050000	0.050000	0.050000	0.050000	0.050000	0.050000	0.050000
	FH-RPs	0.040125	0.031535	0.024455	0.018643	0.013901	0.010068	0.007012	0.004625
	PKM-RPs	0.040127	0.031537	0.024457	0.018645	0.013902	0.010069	0.007013	0.004626
	NTLBG-RPs	0.072800	0.069000	0.063800	0.057400	0.049600	0.041200	0.032000	0.023200
n=25	QMC-RPs	0.040000	0.040000	0.040000	0.040000	0.040000	0.040000	0.040000	0.040000
	FH-RPs	0.047317	0.039514	0.032814	0.027069	0.022149	0.017945	0.014364	0.011327
	PKM-RPs	0.047319	0.039516	0.032817	0.027071	0.022151	0.017947	0.014365	0.011328
	NTLBG-RPs	0.055200	0.055600	0.056200	0.056200	0.055800	0.054800	0.052600	0.049400
n=28	QMC-RPs	0.035714	0.035714	0.035714	0.035714	0.035714	0.035714	0.035714	0.035714
	FH-RPs	0.049638	0.042416	0.036105	0.030598	0.025797	0.021618	0.017984	0.014831
	PKM-RPs	0.049640	0.042418	0.036107	0.030600	0.025800	0.021620	0.017986	0.014833
	NTLBG-RPs	0.045800	0.046000	0.046400	0.047000	0.047800	0.048600	0.048800	0.048800
n=30	QMC-RPs	0.033333	0.033333	0.033333	0.033333	0.033333	0.033333	0.033333	0.033333
	FH-RPs	0.050614	0.043782	0.037752	0.032439	0.027763	0.023651	0.020039	0.016869
	PKM-RPs	0.050616	0.043785	0.037755	0.032442	0.027765	0.023653	0.020041	0.016872
	NTLBG-RPs	0.041000	0.041000	0.041400	0.041800	0.042200	0.043200	0.044000	0.044600
	category	P17	P18	P19	P20	P21	P22	P23	P24
n=20	QMC-RPs	0.050000	0.050000	0.050000	0.050000				
	FH-RPs	0.002817	0.001512	0.000644	0.000154				
	PKM-RPs	0.002818	0.001512	0.000644	0.000154				
	NTLBG-RPs	0.015400	0.009000	0.004400	0.001400				
n=25	QMC-RPs	0.040000	0.040000	0.040000	0.040000	0.040000	0.040000	0.040000	0.040000
	FH-RPs	0.008767	0.006629	0.004862	0.003426	0.002284	0.001405	0.000761	0.000327
	PKM-RPs	0.008769	0.006629	0.004863	0.003426	0.002284	0.001405	0.000761	0.000327
	NTLBG-RPs	0.044800	0.039200	0.032800	0.026000	0.019200	0.012800	0.007400	0.003800
n=28	QMC-RPs	0.035714	0.035714	0.035714	0.035714	0.035714	0.035714	0.035714	0.035714
	FH-RPs	0.012104	0.009754	0.007739	0.006025	0.004579	0.003375	0.002389	0.001600
	PKM-RPs	0.012105	0.009755	0.007740	0.006026	0.004580	0.003376	0.002390	0.001600
	NTLBG-RPs	0.048200	0.046600	0.043600	0.040000	0.035200	0.029400	0.023200	0.017200
n=30	QMC-RPs	0.033333	0.033333	0.033333	0.033333	0.033333	0.033333	0.033333	0.033333
	FH-RPs	0.014094	0.011670	0.009559	0.007730	0.006153	0.004805	0.003662	0.002706
	PKM-RPs	0.014096	0.011672	0.009561	0.007731	0.006154	0.004806	0.003663	0.002707
	NTLBG-RPs	0.045200	0.045400	0.044600	0.043200	0.040600	0.037400	0.032600	0.027600
	category	P25	P26	P27	P28	P29	P30	P31	P32
n=25	QMC-RPs	0.040000							
	FH-RPs	0.000079							
	PKM-RPs	0.000079							
	NTLBG-RPs	0.001200							
n=28	QMC-RPs	0.035714	0.035714	0.035714	0.035714				
	FH-RPs	0.000988	0.000537	0.000231	0.000056				
	PKM-RPs	0.000988	0.000537	0.000231	0.000056				
	NTLBG-RPs	0.011600	0.007000	0.003400	0.001200				
n=30	QMC-RPs	0.033333	0.033333	0.033333	0.033333	0.033333	0.033333		
	FH-RPs	0.001920	0.001289	0.000798	0.000434	0.000188	0.000046		
	PKM-RPs	0.001921	0.001289	0.000798	0.000435	0.000188	0.000046		
	NTLBG-RPs	0.021800	0.016200	0.010800	0.006600	0.003400	0.001200		
